# Multifaceted control of focal points along an arbitrary 3D curved trajectory

**DOI:** 10.1038/s41377-024-01565-4

**Published:** 2024-09-02

**Authors:** Muhammad Afnan Ansari, Hammad Ahmed, Yan Li, Guanchao Wang, Jemma E. Callaghan, Ruoxing Wang, James Downing, Xianzhong Chen

**Affiliations:** 1 Institute of Photonics and Quantum Sciences, School of Engineering and Physical Sciences, Heriot-Watt University, Edinburgh, EH14 4AS UK; 2 School of Materials, Zhengzhou University of Aeronautics, Zhengzhou, 450015 China; 3 School of Physics, Harbin Institute of Technology, Harbin, 150001 China; 4 STMicroelectronics, 1Tanfield, Inverleith Row, Edinburgh, EH3 5DA UK; 5 Department of Mathematics and Physics, North China Electric Power University, Baoding, 071003 China

**Keywords:** Metamaterials, Nanophotonics and plasmonics

## Abstract

Metalenses can integrate the functionalities of multiple optical components thanks to the unprecedented capability of optical metasurfaces in light control. With the rapid development of optical metasurfaces, metalenses continue to evolve. Polarization and color play a very important role in understanding optics and serve as valuable tools for gaining insights into our world. Benefiting from the design flexibility of metasurfaces, we propose and experimentally demonstrate a super metalens that can realize multifaceted control of focal points along any 3D curved trajectory. The wavelengths and polarization states of all focal points are engineered in a desirable manner. The super metalens can simultaneously realize customized 3D positioning, polarization states, and wavelengths of focal points, which are experimentally demonstrated with incident wavelengths ranging from 501 to 700 nm. We further showcase the application of the developed super metalenses in 3D optical distance measurement. The compact nature of metasurfaces and unique properties of the proposed super metalenses hold promise to dramatically miniaturize and simplify the optical architecture for applications in optical metrology, imaging, detection, and security.

## Abstract (web summary)

We demonstrate a metalens that can realize multifaceted control of focal points along any 3D curved trajectory. Each wavelength corresponds to a focal point with a predesigned polarization state.

## Graphical abstract

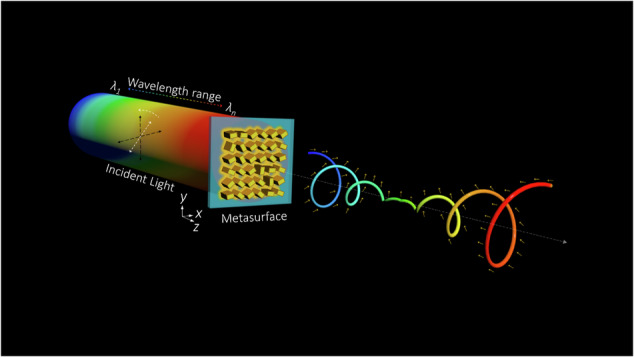

## Introduction

Optical lenses are essential optical elements in optical systems ranging from microscopes to telescopes. Optical lenses are typically designed based on phase accumulation, which is achieved by controlling the surface geometry of optical materials such as glass^1^. However, this approach often results in large volume and limited functionality, failing to align with the prevailing trend of miniaturization and integration. Benefiting from the unprecedented capability of optical metasurfaces in light control^2–10^, optical metalenses are ultrathin and flat, and can be designed to possess multiple functionalities^11–16^. Optical metasurfaces have been used to develop various multifunctional metalenses, including dual-polarity^17^, multi-foci^18–20^, polarization-rotated focal points^21^, spin-selective^22^, dual-mode and dual-wavelength metalenses^6,23^, achromatic metalenses^12,24–27^, and diffraction-limited focusing^13,28–31^. Both polarization and color play a very important role in understanding fundamental optics and serve as valuable tools for gaining insights into our world. With the rapid development of optical metasurfaces, metalenses continue to evolve. Recently, we have developed metalenses to generate various polarization knots^32,33^ and manipulate them in 3D space^34^. However, only a single or several wavelengths are involved in these polarization structures. To accurately map the incident wavelengths into the predesigned focal points located on the same observation plane, a metalens with 180 focal points was used to develop a compact high-resolution spectrometer^35^. It is worth mentioning that all the focal points here are located on the same observation plane and have the same polarization state. There exists a question of whether we can develop a metalens with an extraordinary capability (a super metalens) to realize the multifaceted control of focal points along an arbitrary 3D curved trajectory. The polarization states and wavelengths on these focal points can be controlled in a desirable manner. There are more degrees of freedom for the lens design if polarization states and wavelengths can be customized on arbitrarily distributed focal points in 3D space. In addition, a device that combines multiple functions into a single unit is preferred for applications where there is a need for compactness and integration. It is fundamentally and technically challenging to realize it with conventional optics. To our best knowledge, such a super metalens has not been reported.

To tackle the abovementioned challenges, we propose a super metalens that can realize multifaceted control of focal points along an arbitrary 3D trajectory. The wavelength and polarization state of each focal point are meticulously crafted. The super metalens can generate customized focal points with predesigned polarization states along an arbitrary 3D continuous curve by continuously varying the wavelength of an incident light beam. In addition, the predesigned polarization states can be further modulated by controlling the incident linear polarization. The proposed super metalens is designed with a multi-foci metalens model that can simultaneously control phase, polarization, and wavelength. Unlike previous works^18,21,32–34^, the design incorporates a wide range of operating wavelengths and the independent control of polarization states on these focal points. The multifaceted control of focal points is used to realize 3D positioning and measure the coordinates of these focal points in 3D space. Our approach not only dramatically increases the information capacity through the simultaneous control of polarization and wavelengths, but also leads to miniaturized multifunctional devices. The unique properties of the developed super metalenses may find applications in measurement, detection, security, display, and imaging.

## Results

**Fig. 1:**
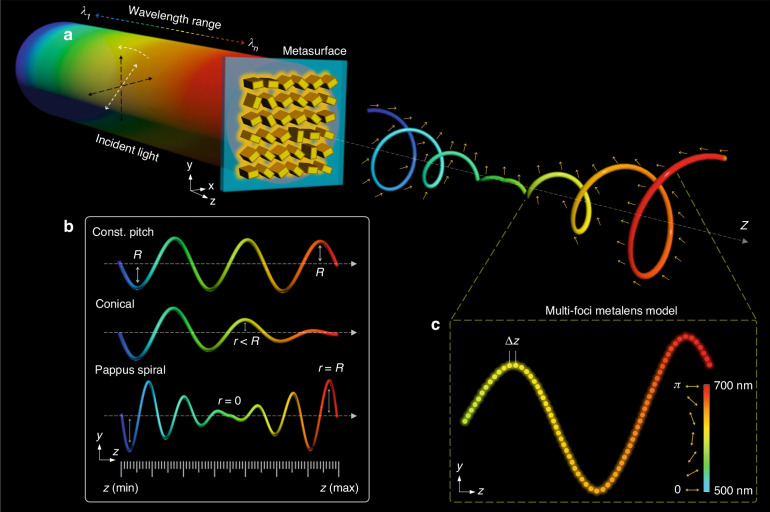
A super metalens for the multifaceted control of focal points along any 3D curved trajectory. **a** Schematic of the proposed super metalens for generating focal points with customized linear polarization states along a 3D Pappus spiral. When the metalens is illuminated with a linearly polarized light beam with continuously variant wavelengths, the focal points with predesigned linear polarization states are generated along the Pappus spiral. The linear polarization directions of focal points (denoted with orange arrows) can be dynamically modulated by continuously changing incident linear polarization. The color gradient of focal points on the transmission side represents the continuous change in the wavelength with a step size of 1 nm. **b** Different 3D spiral curves with perspective on *yz*-plane, including a cylindrical helix with a constant pitch, a conical spiral, and a Pappus spiral. The radius *R* remains constant in the first case, while it varies in the second and third cases. **c** A magnified view of a segment of the 3D curve shown in (**b**). The designed focal points in a 3D space include a continuous wavelength range and polarization rotation angles. Each wavelength corresponds to a focal point. The compression of a 3D spiral is controlled with the number of focal points and longitudinal separation *Δz*. The color bar and orange arrows represent the operating wavelength range (500–700 nm) and linear polarization rotation angle range (0–*π*), respectively

Figure 1 shows the schematic diagram of a proposed super metalens for the multifaceted control of focal points. When the super metalens is illuminated with a linearly polarized (LP) incident light beam with continuously variant wavelengths, the generated focal points are located on a customized 3D curved trajectory (Fig. 1a). Each incident wavelength corresponds to a focal point, whose polarization state is independently controlled through the polarization rotation with respect to the incident linear polarization. A predesigned 3D continuous curve with customized polarization information is generated on the transmission side by continuously changing the incident wavelengths ranging from 501 to 700 nm with a step size of 1 nm. More interestingly, the designed polarization state on each focal point can be further modulated by changing the incident linear polarization.

To demonstrate the multifaceted control of focal points along arbitrary 3D trajectories, we choose various 3D spiral curves, including a cylindrical helix with a constant pitch, a conical spiral, and a Pappus spiral (Fig. 1b). A multi-foci metalens model is used to design the super metalens. Our design includes multiple variables such as longitudinal distance (*z*), longitudinal separation (*Δz*), number of focal points (*N*), wavelengths (*λ*), and polarization rotation angles (*Γ*), as illustrated in Fig. 1c. The designed super metalens is realized with a geometric metasurface, which consists of gold nanorods with spatially variant orientations sitting on an indium tin oxide (ITO) coated glass substrate.

We start with a metalens that can generate an off-axis focal point at a wavelength *λ*. The desired phase profile is governed by:

1 $$\varphi \left(x,y\right)=-\frac{2\pi }{\lambda }\left(\sqrt{{f}^{2}+{\left(x-{x}_{0}\right)}^{2}+{\left(y-{y}_{0}\right)}^{2}}-{f}_{D}\right)$$

where (*x*_*0*_, *y*_*0*_, *f*) denote the coordinates of the focal point in 3D space and *z* = *f* is the position of the focal plane. ${f}_{D}=\sqrt{{x}_{0}^{2}+{y}_{0}^{2}+{f}^{2}}$ is defined as the distance between a focal spot and central point of the metalens. A continuous 2D focal curve can be generated by increasing the number of focal points^32^. The desired phase profile of geometric metasurfaces can be reformulated through the phase multiplexing^4^ as:

2 $${\varphi }_{n}\left(x,y\right)={\rm{arg }}\left\{\mathop{\sum }\limits_{n=1}^{N}{e}^{i\left[-\frac{2\pi }{\lambda }\left(\sqrt{{f}^{2}+{\left(x-{x}_{n}\right)}^{2}+{\left(y-{y}_{n}\right)}^{2}}-\sqrt{{x}_{n}^{2}+{y}_{n}^{2}+{f}^{2}}\right)\right]}\right\}$$

It is worth mentioning that the above multi-foci metalens is designed for circular polarization (CP) states, i.e., left circular polarization (LCP) or right circular polarization (RCP). To realize polarization rotation at each focal point under the illumination of LP light, the phase profile is modified as:

3 $$\begin{array}{l}{\varphi }_{n}\left(x,y\right)={\rm{arg }}\left\{\mathop{\sum }\limits_{n=1}^{N}\left({e}^{i\left[-\frac{2\pi }{\lambda }\left(\sqrt{{f}^{2}+{\left(x-{x}_{n}\right)}^{2}+{\left(y-{y}_{n}\right)}^{2}}-\sqrt{{x}_{n}^{2}+{y}_{n}^{2}+{f}^{2}}\right)+{\varGamma }_{n}\right]}\right.\right.\\\qquad\qquad\left.\left.{+\,e}^{-i\left[-\frac{2\pi }{\lambda }\left(\sqrt{{f}^{2}+{\left(x-{x}_{n}\right)}^{2}+{\left(y-{y}_{n}\right)}^{2}}-\sqrt{{x}_{n}^{2}+{y}_{n}^{2}+{f}^{2}}\right)-{\varGamma }_{n}\right]}\right)\right\}\end{array}$$

*Γ*_*n*_ represents the additional control variable for the polarization rotation in comparison with the direction of the incident linear polarization. In Eq. 3, the phases carrying terms ${e}^{i\varphi \left(x,y\right)}$ and ${e}^{-i\varphi \left(x,y\right)}$ are responsible for converging LCP and RCP components of the light beam, respectively. Because an LP light beam can be decomposed into LCP and RCP with equal components, the superposition of LCP and RCP components can generate a polarization rotation angle *Γ*_*n*_ at a predesigned focal point. There are four CP components involved in the output light beam with different phase terms, however, only two CP components carry the desired phase profiles to generate the focal points with the required polarization rotation profiles. A detailed explanation is available in the ref. ^21^.

For the design presented in Eq. 3, all focal points appear on the same observation plane at a single working wavelength *λ*. Going further than previous attempts, which concentrated on just one or several working wavelengths^21,32,34^, we first include a super wavelength-multiplexed functionality by incorporating a new control variable *λ*_*n*_. Consequently, the phase profile in Eq. 3 can be modified as:

4 $$\begin{array}{l}{\varphi }_{n}\left(x,y\right)={\rm{arg }}\left\{\mathop{\sum }\limits_{n=1}^{N}\left({e}^{i\left[-\frac{2\pi }{{\lambda }_{n}}\left(\sqrt{{f}^{2}+{\left(x-{x}_{n}\right)}^{2}+{\left(y-{y}_{n}\right)}^{2}}-\sqrt{{x}_{n}^{2}+{y}_{n}^{2}+{f}^{2}}\right)+{\varGamma }_{n}\right]}\right.\right.\\\qquad\qquad\left.\left.{+\,e}^{-i\left[-\frac{2\pi }{{\lambda }_{n}}\left(\sqrt{{f}^{2}+{\left(x-{x}_{n}\right)}^{2}+{\left(y-{y}_{n}\right)}^{2}}-\sqrt{{x}_{n}^{2}+{y}_{n}^{2}+{f}^{2}}\right)-{\varGamma }_{n}\right]}\right)\right\}\end{array}$$

where *N* also denotes the value of the final index for the wavelength control variable. With the formulation provided in Eq. 4, each focal point corresponds to a specific wavelength. Furthermore, different from the previous design in wavelength multiplexing^32,33,35^, we introduce an additional control variable to regulate the longitudinal position (z coordinates) along the light propagation direction. The desired phase profile of the super metalens is governed by:

5 $$\begin{array}{l}{\varphi }_{n}\left(x,y\right)={\rm{arg }}\left\{\mathop{\sum }\limits_{n=1}^{N}\left({e}^{i\left[-\frac{2\pi }{{\lambda }_{n}}\left(\sqrt{{z}_{n}^{2}+{\left(x-{x}_{n}\right)}^{2}+{\left(y-{y}_{n}\right)}^{2}}-\sqrt{{x}_{n}^{2}+{y}_{n}^{2}+{z}_{n}^{2}}\right)+{\varGamma }_{n}\right]}\right.\right.\\\qquad\qquad\left.\left.{+\,e}^{-i\left[-\frac{2\pi }{{\lambda }_{n}}\left(\sqrt{{z}_{n}^{2}+{\left(x-{x}_{n}\right)}^{2}+{\left(y-{y}_{n}\right)}^{2}}-\sqrt{{x}_{n}^{2}+{y}_{n}^{2}+{z}_{n}^{2}}\right)-{\varGamma }_{n}\right]}\right)\right\}\end{array}$$

where *z*_*n*_ represents the *z* coordinate of the *n*th focal point. Depending on the chosen trajectory in 3D space, the super metalens design in Eq. 5 integrates all necessary control variables to achieve the multifaceted control of focal points.

**Fig. 2:**
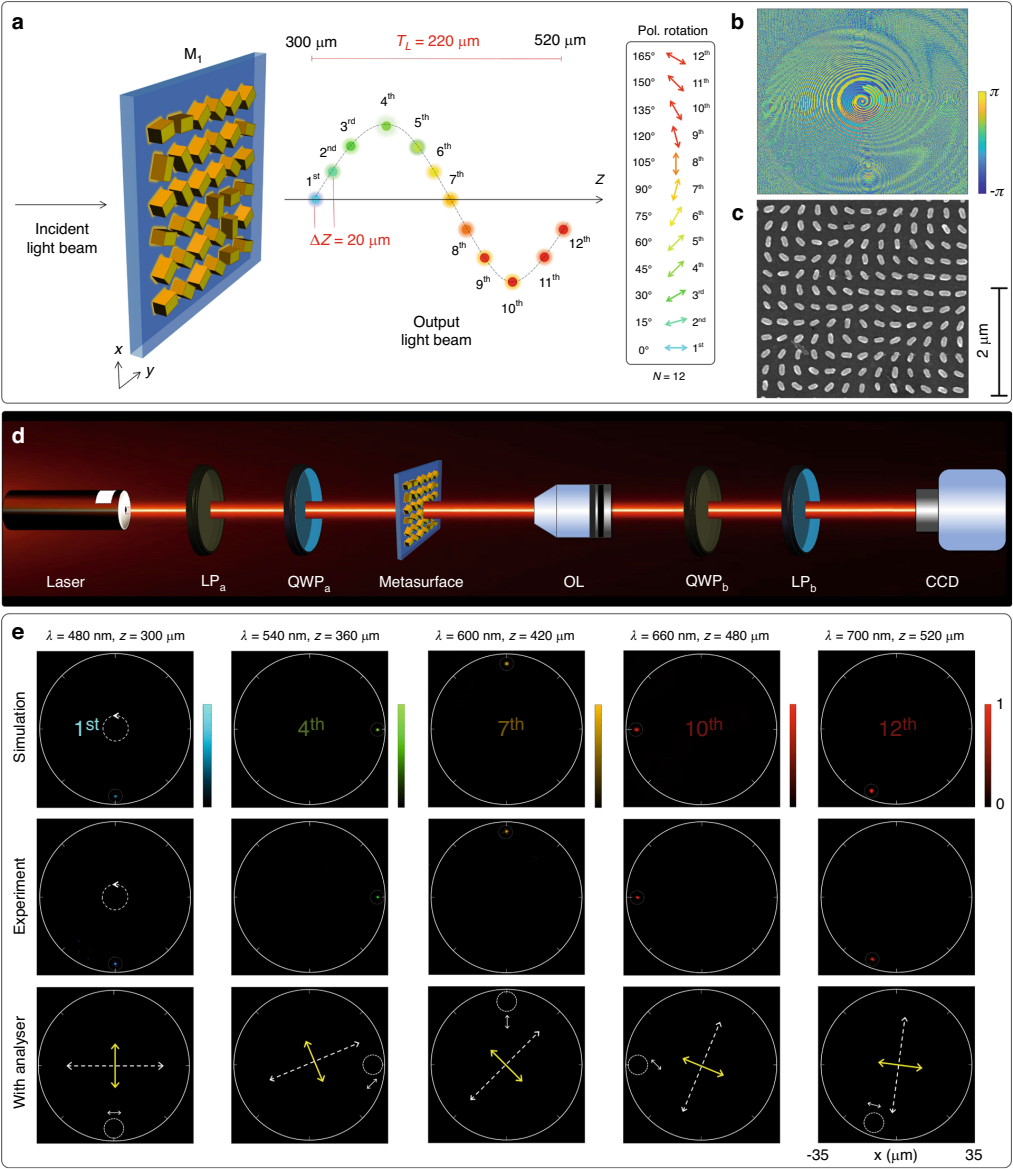
Design, fabrication, and characterization of a super metalens with 12 focal points. **a** The schematic of a super metalens M1 with 12 focal points on a 3D cylindrical helix trajectory. The right inset shows the values of the polarization rotation angles. The color gradient represents the operating wavelengths ranging from 480 to 700 nm with a step size of 20 nm. The total longitudinal length *T*_*L*_ of the cylindrical helix is 220 µm, which spans from 300 to 520 µm. The longitudinal separation *Δz* between the focal points is a constant and equal to 20 µm. **b** The total phase profile of the proposed super metalens. **c** An SEM image of the fabricated metasurface consisting of gold nanorods. **d** The schematic of an optical setup for characterizing the super metalens. LP_a_ and LP_b_: linear polarizers, QWP_a_ and QWP_b_: quarter-wave plates, OL: ×50 objective lens, CCD: charge-coupled device. For results with LP incident light beam, both quarter-wave plates are removed from the optical setup. **e** Intensity distributions of the selected focal points (1st, 4th, 7th, 10th, and 12th focal spots) along the 3D cylindrical helix path at various operating wavelengths (480, 580, 600, 660, and 700 nm) and longitudinal distances (300, 360, 420, 480, and 520 µm). Under the illumination of the RCP incident light beam, the first and second rows show the simulation and experimental results, respectively. The third row shows the modulated intensity distributions with an analyzer, which are used to indirectly confirm the designed polarization rotation angles under the illumination of incident LP light. The regions of corresponding focal points are highlighted with dotted white circles. The constant radius of the cylindrical helix is shown with solid white circles as references. The dashed white arrows and solid yellow arrows represent the direction of incident polarization and the transmission axis of the analyzer, respectively. The solid white arrows with the white-dotted circles show the initial polarization rotation angles (at *β* = 0°) for each focal point

We begin by designing a super metalens M_1_ that can generate 12 focal points along the longitudinal direction with a uniform increment, as illustrated in Fig. 2a. In this design, the curved trajectory is a circular cylindrical helix with a constant pitch and a fixed radius, governed by the following parametric equations:

6 $$\left\{\begin{array}{c}{x}_{n}=R\sin ({C\theta }_{n})\\ {y}_{n}=R\cos ({C\theta }_{n})\\ {z}_{n}={z}_{i}+(n-1)\varDelta z\end{array}\right.$$

where *R* = 30 µm and *θ*_*n*_ represent the radius and polar angles of the circular cylindrical helix, respectively. *C* = 1 is a constant positive parameter that controls the number of cycles in the cylindrical helix. The variables *Δz* and *z*_*i*_ are used to control longitudinal separation and initial longitudinal distance, respectively. With a fixed longitudinal separation *Δz* set at 20 µm, the total longitudinal length of the trajectory (*T*_*L*_) is equal to 220 µm, which spans from 300 to 520 µm. The wavelength of each focal point is governed by the wavenumber expression ${k}_{n}=2\pi /{[\lambda }_{i}+(n-1)\varDelta \lambda ]$, where the initial wavelength *λ*_*i*_ and the wavelength separation *Δλ* are 480 and 20 nm, respectively. As shown in the right panel of Fig. 2a, all designed focal points on the circular cylindrical helix carry distinct linear polarization rotations. The calculated total phase profile of the metasurface M_1_ based on Eq. 5 is provided in Fig. 2b.

The desired phase profile is realized with a geometric metasurface consisting of gold nanorods with spatially variant orientation angles governed by ${\varphi }_{n}\left(x,y\right)/2$. The length, width, and height of each nanorod are 220, 130, and 40 nm, respectively. The pixel size is 300 nm along both the *x* and *y* directions. Each metasurface has an area of 400 × 400 µm^2^. The metasurface exhibits a broadband response, covering the wavelength range of 500–700 nm. The transmission spectrum is provided in Supplementary Section 1. The low efficiency of the proposed plasmonic metasurface is due to the high nonconverted part, which can be resolved by using a dielectric metasurface with high efficiency in the visible domain^36–39^. The metasurface samples are fabricated using standard electron-beam lithography, gold film deposition, and lift-off process. The scanning electron microscope image of the metasurface with a scale bar of 2 µm is shown in Fig. 2c. The metalens is experimentally characterized with an optical setup shown in schematic Fig. 2d. Detailed fabrication process and the experimental setup are available in the Experimental Details section.

The presence of focal points along the predesigned trajectory is confirmed by observing intensity patterns at the designated locations in the observation plane, which is realized by concurrently varying the corresponding wavelengths and longitudinal distances under the illumination of the CP incident light beam. The simulated and experimental intensity patterns at various wavelengths and longitudinal distances are provided in the first and second rows of Fig. 2e, respectively. In Fig. 2e, only 5 out of 12 focal points are shown, i.e., 1st, 4th, 7th, 10th, and 12th focal points. The wavelength ranges from 480 to 700 nm, while the longitudinal distance ranges from 300 to 520 µm. The selection of five focal points is based on their positions on the circular cylindrical helix to better illustrate the complete coverage of the designed trajectory. Detailed results for all 12 focal points can be found in Supplementary Section 2. In each plot, solid white circles mark the boundary of the circular cylindrical helix. Additionally, dashed white circles indicate the positions of the corresponding focal points, accompanied by dashed white circles with arrows to illustrate the helicity of the CP incident light beam. Both simulation and experimental results confirm the accurate mapping of focal points onto the circular cylindrical helix trajectory. Furthermore, the independent polarization rotation angles of focal points along the optical path are verified by examining the dark intensity patterns with an analyzer (linear polarizer) under the illumination of incident LP light. The transmission axis of the analyzer is perpendicular to the incident polarization direction, as shown in the third row of Fig. 2e. Here, the polarization direction of the LP incident light beam has an inclined angle *β* with respect to the *x*-axis. The dark region in the measured intensity profile corresponds to the predesigned polarization rotation that is equal to 2*β* or 2*β* + *π*. As an illustration, the fourth focal point corresponds to a wavelength of 540 nm and a longitudinal distance of 345 µm. The polarization rotation angle for this focal point is 45° under the illumination of a horizontally polarized incident light beam. To verify the polarization state of 45°, the angle of the LP incident beam and the analyzer are adjusted to 22.5° and 112.5°, respectively. The presence of dark intensity patterns confirms the predesigned polarization rotation angles of focal points, as depicted in the third row of Fig. 2e. The white dashed arrows and yellow solid arrows in Fig. 2e indicate the directions of the transmission axes of the linear polarizer and analyzer, respectively. The solid white arrows near the dashed white circles show the directions of initial polarization rotation angles of the design M_1_ under the illumination of the horizontally polarized light beam (*β* = 0°). The simulation results of the proposed super metalens are obtained using the Fresnel–Kirchhoff diffraction integral method^32,38^.

**Fig. 3:**
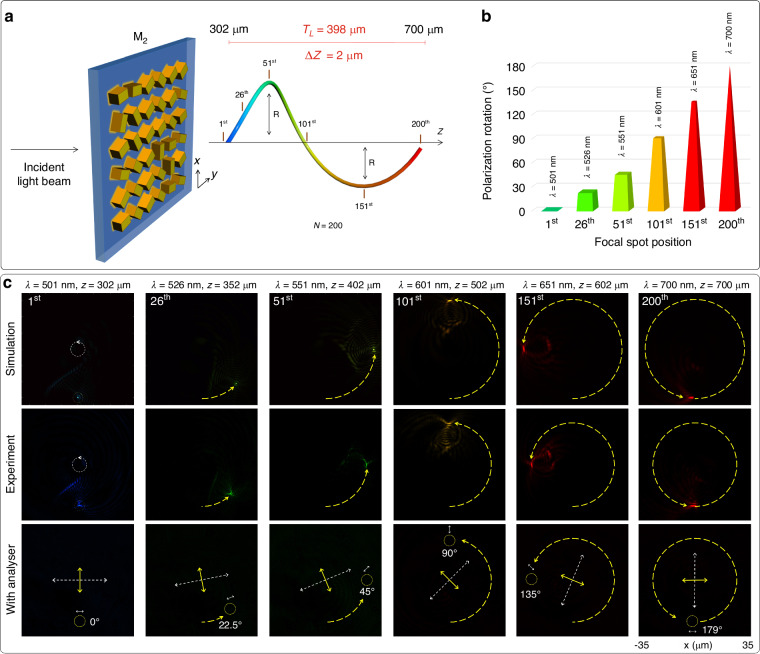
A super metalens with 200 focal points located along a single-cycle 3D cylindrical helix. **a** The schematic diagram of super metalens M_2_ with focal points on a continuous single-cycle 3D cylindrical helix trajectory with a constant pitch and a constant radius. There are 200 operating wavelengths ranging from 501 to 700 nm with a step size of 1 nm. The longitudinal separation is equal to 2 µm, which makes the total longitudinal length of the cylindrical helix equal to 398 µm, spanning from 302 to 700 µm. **b** The polarization rotation angles and operating wavelengths of the selected focal points. **c** Intensity distributions of the selected focal points (1st, 26th, 51st, 101st,151st, and 200th) at the corresponding operating wavelengths (501, 526, 551, 601, 651, and 700 nm) and longitudinal distances (302, 352, 402, 502, 602, and 700 µm). The direction of the analyzer (solid yellow arrow) is always perpendicular to the polarization direction of the incident light beam (dashed white arrow). The dashed yellow curves with arrows represent the mapped trajectory by the focal points on the single-cycle 3D cylindrical helix with a constant radius. The solid white arrows with dotted yellow circles represent the initial polarization rotation angles (at *β* = 0°). The regions of the corresponding focal points are highlighted with dotted yellow circles

Next, we increase the number of focal points to create a continuous circular cylindrical helix trajectory. The focal depth of the designed focal point is ~8 µm, and the maximum intensity is observed at the designated longitudinal distance *z*_*n*_. The detailed analysis of the focal point, accuracy, spot profile, and full width at half maximum (FWHM) is provided in Supplementary Section 3. The effect of the aperture size of the metalens on the focal spot is discussed in Supplementary Section 4. The variation in the focal spot size for different longitudinal distances is minimized by optimizing the longitudinal separation. In the design of the super metalens (M_2_), a longitudinal separation of 2 µm is chosen with an increased number of focal points (*N* = 200). The trajectory of the continuous circular cylindrical helix is governed by Eq. 6, with *C* = 1 for a single cycle. Given that the longitudinal separation is approximately four times less than the focal depth, the intensity patterns of the focal points partially overlap, generating a near-continuous trajectory. Supplementary Section 5 further discusses the scenarios with low and high values of longitudinal separation (*Δz* = 1 and 5). Figure 3a shows the schematic of design M_2_, where the radius is a constant (*R* = 30 µm), and the 200 focal points have distinct polarization rotation angles that vary linearly from 0° to 180°. Six distinct positions are chosen to demonstrate the mapping of focal points. The polarization rotation angles, and their corresponding wavelengths are depicted in Fig. 3b. The focal points can be continuously mapped onto the predesigned trajectory by simultaneously sweeping the wavelength and longitudinal distance, as illustrated in Fig. 3c. The yellow curved arrows are used to identify the presence of focal points along the 3D curved trajectory. The first two rows illustrate the intensity profiles and the presence of focal points under the illumination of the RCP light beam through both simulation and experimental measurement. The final row shows the experimental results with the analyzer under the illumination of LP incident light, confirming the presence of unique predesigned polarization rotations. The values given to the dashed yellow circles indicate the initial polarization rotation angles of each focal point when *β* = 0°. A detailed analysis of focal points with the cross-sectional distribution along the *z*-axis is presented in Supplementary Section 6. Both simulation and experimental results confirm that the proposed super metalens M_2_ can accurately map the focal points onto the predesigned trajectory of the continuous circular cylindrical helix with on-demand polarization rotation angles. The focusing efficiency of the super metalens is discussed in Supplementary Section 7.

**Fig. 4:**
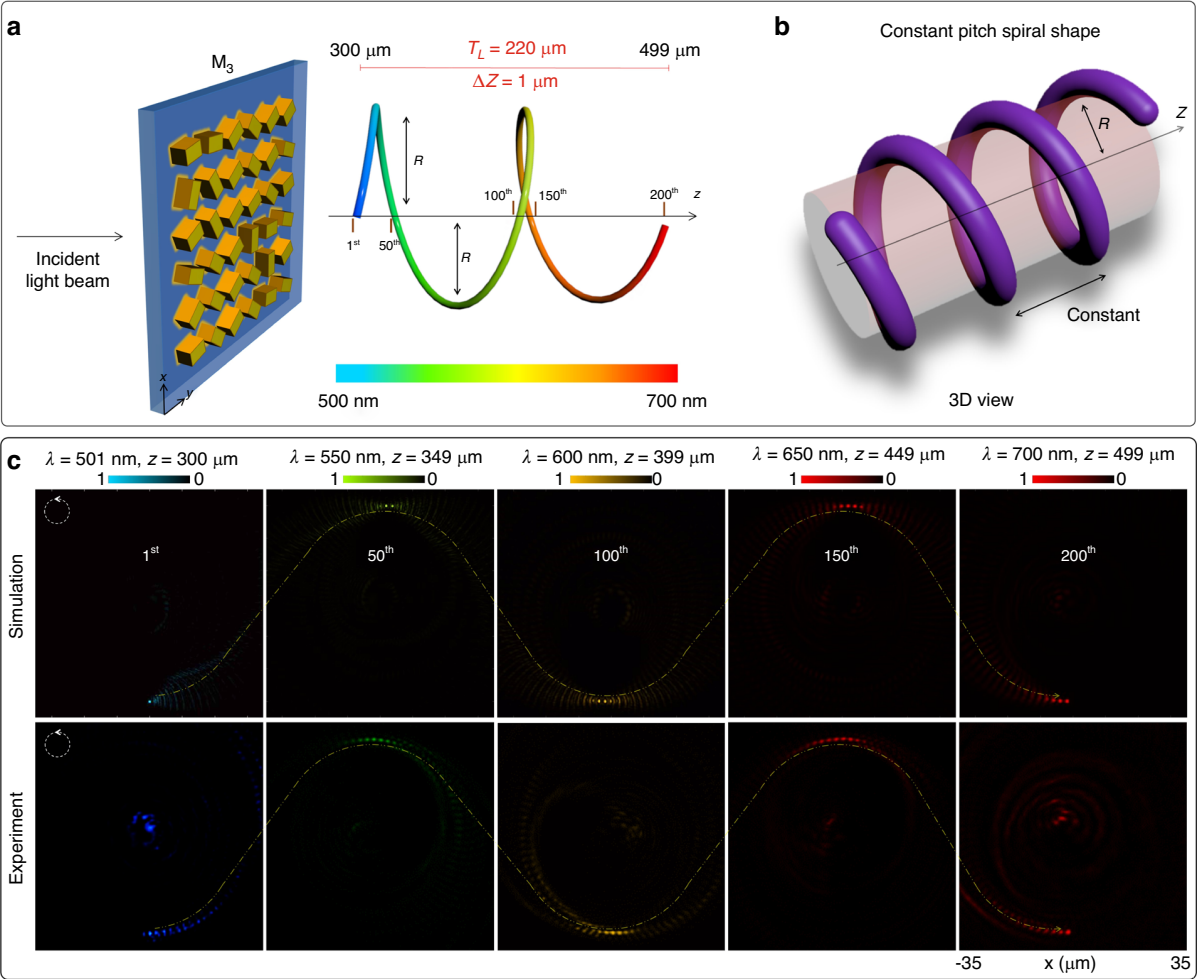
Focal points along a double-cycle cylindrical helix. **a** The schematic diagram of the super metalens M_3_ for generating 200 focal points along a double-cycle continuous 3D cylindrical helix with constant pitch and radius. The longitudinal separation is 1 µm, which makes the total length of the curved trajectory equal to 200 µm. The first and last focal points are located at *z* = 300 µm and *z* = 499 µm, respectively. **b** The 3D view of the spiral shape with a constant pitch and a constant radius. **c** The simulation and measured results of the intensity distributions of selected focal points (1st, 50th, 100th, 150th, and 200th) under the illumination of RCP incident light beam at corresponding operating wavelengths (501, 550, 600, 650, and 700 nm) and longitudinal distances (300, 349, 399, 449, and 499 µm). The dashed yellow curved path shows the antinodes of the *y*-axis along the longitudinal distance. It is used to indicate the positions of the generated focal points at the desired locations in 3D space

The third super metalens M_3_ (Fig. 4a), inspired by a spring shape (Fig. 4b), is designed with two cycles (*C* = 2) and increased compression (*Δz* = 1). The total length of the spring-shaped trajectory is *T*_*L*_ = 200 µm. The wavelength range spans from 501 to 700 nm with a step size of 1 nm, as depicted in Fig. 4a with a color bar. The remaining parameters, such as polarization rotation angles, are consistent with the previous design. Figure 4c showcases the simulation and experimental results for the selected points under the illumination of the RCP incident light beam, namely the 1st, 50th, 100th, 150th, and 200th points. These selected points are precisely aligned with the antinodes of the *y*-axis in 3D space, as indicated by the dashed yellow curved path. It is evident that the proposed super metalens M_3_ accurately maps the focal points onto two cycles of the spring-shaped trajectory in 3D space. This occurs under the illumination of wavelengths ranging from 501 to 700 nm at their corresponding longitudinal distances. Some adjacent focal points are also visible alongside the desired focal point due to the intrinsic dispersion of the incident wavelength at other designed focal positions. Consequently, the focal point with the highest intensity corresponds to the position of interest. The impact of the intrinsic dispersion of the incident wavelength at other designed focal positions is discussed in Supplementary Section 8. The effect of the number of focal points and longitudinal separation on the level of crosstalk is discussed in Supplementary Section 9. The unwanted noise observed in the experiment is also due to the nonconverted part of the CP incident light beam and fabrication errors.

**Fig. 5:**
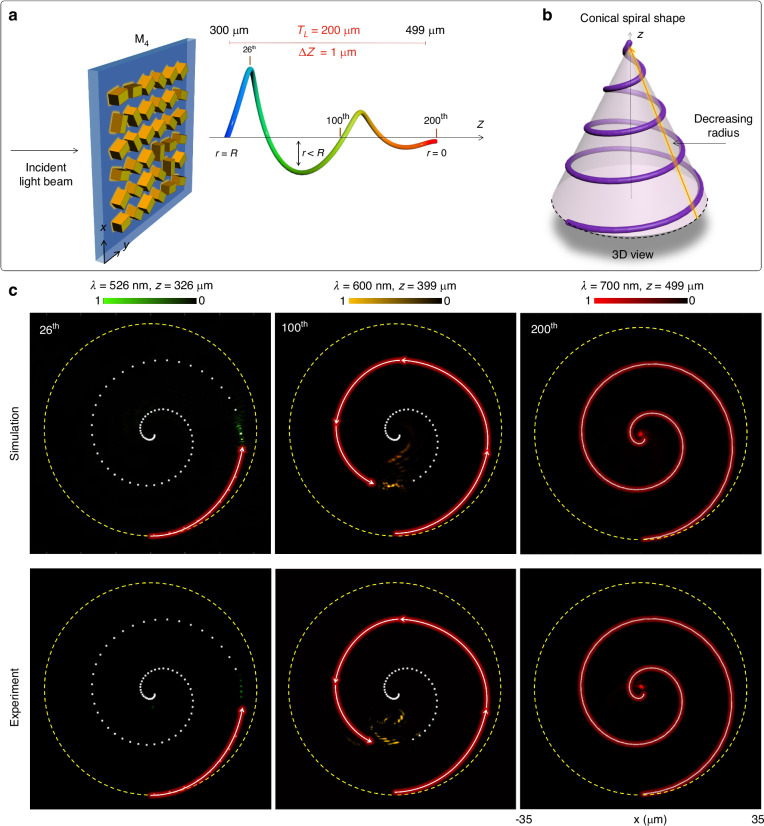
Focal points along a 3D conical spiral trajectory. **a** The schematic diagram of the super metalens M_4_ for generating 200 focal points on a continuous 3D conical spiral trajectory with a decreasing radius. The maximum and minimum radii are 30 µm and 0, respectively. The wavelength is modulated with an operating range spanning from 501 to 700 nm with a step size of 1 nm. **b** The 3D view of the conical spiral shape with a decreasing radius. **c** The simulated and measured intensity distributions of the designed super metalens for the selected focal points on a 3D conical spiral trajectory under the illumination of an RCP incident light beam. The results for the selected focal points (26th, 100th, and 200th) are acquired at the operating wavelengths (526, 600, and 700 nm) and longitudinal distances (326, 399, and 499 µm). The dashed yellow circles represent the maximum and initial value of the radius. The solid red portion and dotted white portion of spiral curves are used to illustrate the evolution of the generated focal points along the curved trajectory of a conical spiral with a decreasing radius in 3D space

In all the preceding designs, the radius of the spiral structures is a constant. However, unique spiral paths can be generated by incorporating variable radius in the design of the super metalens, as illustrated in Fig. 5a. Consequently, our subsequent super metalens M_4_ draws inspiration from the 3D conical spiral with a decreasing radius along the longitudinal direction, as depicted in Fig. 5b. The curved trajectory of a conical spiral is determined by parametric Eq. 6 with *C* = 2 and a variable radius *R* = *r*_*n*_, where ${r}_{n}={R}_{i}-(n-1)\varDelta R$ is a function with an initial value of radius *R*_*i*_ and an incremental shift *ΔR*. The initial and final values of radius are 30 µm and 0, respectively. Figure 5c shows the simulation and experimental results of super metalens M_4_, accurately mapping the focal points onto a 3D optical trajectory governed by a conical spiral shape by controlling the wavelength and the longitudinal observation plane. For illustrative purposes, three points on the optical path are selected to demonstrate the concept, namely the 26th, 100th, and 200th points. The initial value of the radius is depicted in a dashed yellow circle as a reference. The radius for the 26th focal point is slightly less than the initial radius value, which is confirmed through the appearance of a focal point with the highest intensity at the position designed for 526 nm. According to design M_4_, the value of the radius is halved at 600 nm, resulting in the focal point with the highest intensity located in the middle of the conical spiral trajectory. At the end of the conical spiral (*z* = 499 µm), the focal point with the highest intensity is located in the middle with the minimum value of the radius at 700 nm. The red-curved arrows and white-dotted curves in Fig. 5c represent the mapped and unmapped trajectories of a conical spiral, respectively.

**Fig. 6:**
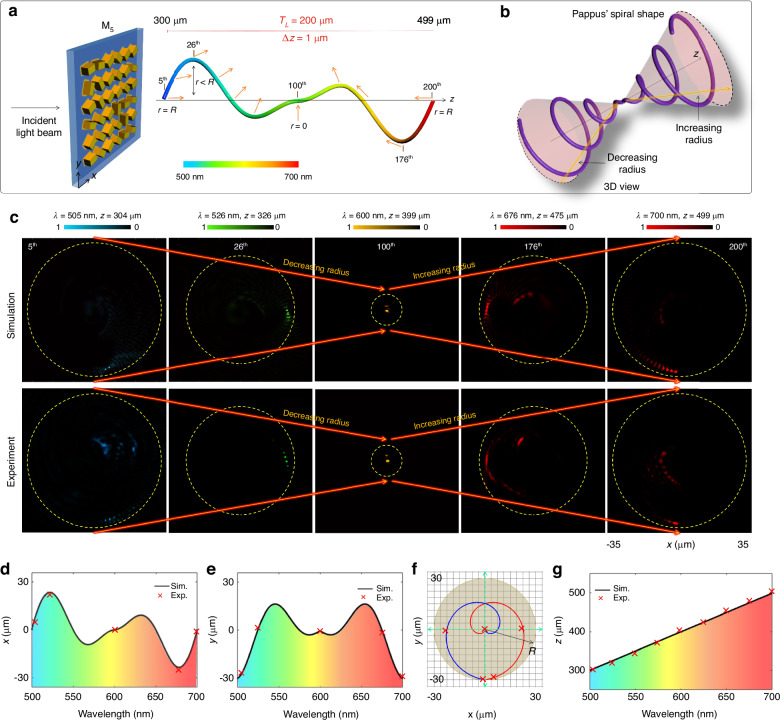
A super metalens with focal points along a 3D Pappus spiral trajectory and its application in 3D optical distance measurement. **a** The schematic diagram of the super metalens M_5_ with 200 focal points on a continuous 3D Pappus spiral trajectory. The designed trajectory has a decreasing and increasing radius profile with a maximum value of radius at the initial and final positions of the Pappus spiral. The minimum value of the radius is at the 100th focal point. Unique linear polarization rotation angles (orange arrows) ranging from 0 to *π* are modulated independently for each focal point. **b** A 3D view of the Pappus spiral shape with decreasing and increasing radius profile. **c** The simulated and measured intensity distributions of the designed super metalens for selected focal points along a 3D Pappus spiral trajectory under the illumination of an RCP incident light beam. The results for the selected focal points (5th, 26th, 100th, 176th, and 200th) are acquired at the operating wavelengths (505, 526, 600, 676, and 700 nm) and the longitudinal distances (304, 326, 399, 475, and 499 µm). The decreasing and increasing dashed yellow circles are drawn for references to show the corresponding change in the radius along the optical path. **d**–**g** Coordinates measurement of a focal point with the proposed super metalens. The trajectory of the focal points as a function of wavelength in (**d**) *x*, (**e**) *y*, and (**g**) *z* directions. The solid black curves and red markers represent the simulated and measured values, respectively. **f** An illustration of focal points along the trajectory in *xy*-plane. The portions of the trajectory corresponding to the decreasing and increasing radii are plotted with solid red and blue curves, respectively. The red cross markers show the measured coordinates of the unknown focal points in 3D space (*x*, *y*, *z*)

Similarly, a super metalens can be conceptualized for an inverted 3D conical spiral trajectory, featuring an increasing radius, which is elaborated in Supplementary Section 10. The design M_4_, characterized by a decreasing radius, and its counterpart with an increasing radius can be amalgamated to generate a more intricate 3D trajectory, as depicted in Fig. 6a. The innovative design of the super metalens, denoted as M_5_, takes inspiration from a 3D Pappus spiral, showcasing both decreasing and increasing radii along the longitudinal direction, as illustrated in Fig. 6b. In this design, variables such as wavelengths and polarization rotation angles are incorporated, spanning from 501 to 700 nm and 0° to 179.1°, respectively, encompassing 200 focal points. The color bar and orange arrows in Fig. 6a denote the wavelengths and directions of polarization rotation angles for the 200 focal points, respectively. The coordinates of each point on the 3D Pappus spiral trajectory are determined by parametric Eq. 6, featuring a modified radius function *R* = *r*_*n*_. The expression for the modified *r*_*n*_ can be represented as:

7 $${r}_{n}=\left\{\begin{array}{ll}{R}_{i}-\left(n-1\right)\varDelta R & {\rm{if}}1\le n\le 99\\ \left(n-100\right)\varDelta R & {\rm{if}}100\le n\le 200\end{array}\right.$$

where the value of *ΔR* is 0.3 µm. To illustrate the impact of variable radius on the 3D trajectory governed by the Pappus spiral, five specific points (5th, 26th, 100th, 176th, 200th) are selected. The simulation and experimental results validate the conceptualization of design M_5_, showcasing an accurate mapping of focal points onto the Pappus spiral trajectory, as illustrated in Fig. 6c. Dashed yellow circles with different radii are drawn to show the new positions of focal points along the 3D trajectory with decreasing and increasing radii. According to Eq. 7, the radius reaches its maximum at *n* = 1 and *n* = 200 and gradually diminishes to its minimum value at *n* = 100. Consequently, a focal point with the highest intensity appears at the maximum value of the radius at both the beginning and end of the Pappus spiral trajectory, as predicted by Eq. 7. On the other hand, the focal point with the highest intensity is observed in the middle, exhibiting the minimum value of the radius at *λ* = 600 nm and *z* = 399 µm. Intensity distributions are measured within the range of minimum and maximum values of the radii. These further substantiate the precise mapping of the focal points onto the desired trajectory in 3D space. The polarization rotation angles of the focal points are experimentally confirmed by observing the dark intensity regions, which are discussed in Supplementary Section 11. Figure 6a–c validate that the proposed super metalens adeptly maps the focal points onto a complex continuous trajectory in 3D space at the corresponding wavelengths and the longitudinal distances. The effect of different 3D curved trajectories on the performance of the super metalens is discussed in Supplementary Section 12.

## Discussion

The multifaceted control of focal points along various predesigned 3D curved trajectories is based on the metasurface design with more control variables such as compression (*N*, *Δz*), wavelength (*λ*), longitudinal distance (*z*), polarization rotation angle (*Γ*) and position coordinates (*x*, *y*), providing more design flexibility. Unlike previous works^32–34,39^, where the design is based on a single or several wavelengths, the extraordinary capability of the super metalenses enables the multiple focal points to have predesigned polarization states along various arbitrary 3D curved trajectories by continuously changing the incident wavelength ranging from 501 to 700 nm. A detailed comparison of differentiated functionalities, design methodologies, and applications of this work and previous works is provided in Supplementary Section 13. The demonstrated 3D positioning, polarization states, and wavelengths of focal points can dramatically increase the information capacity of the metadevice and perform extremely complex optical tasks that are not possible with conventional optics.

The unusual properties of advanced multifunctional metalenses offer extraordinary capabilities beyond traditional lenses and pave the way for more compact optical devices in the future, which can find new applications. The generated 3D curved trajectories are used to measure the coordinates of a focal point in 3D space or wavelength of the incident light beam, as shown in Fig. 6d–g. To measure the coordinates of an unknown and arbitrary focal point, two focal points are selected as two reference points. For example, the first and final focal points on the Pappus spiral are selected as the reference points. The coordinates of unknown and arbitrary focal points in *x*, *y*, and *z* directions can be obtained based on the measured intensity distribution of the highest intensity focal point. For example, the measured optical distances of the highest intensity focal point from the center at *λ* = 526 nm are obtained with values equal to 22.4 and 0.13 µm in *x* and *y* directions, as shown in Fig. 6d, e, respectively. The solid black curve and red cross marker show the simulated and measured results of the optical distances. The simulated and measured coordinates of design M_5_ are also mapped onto a 2D plot in *xy*-plane, which confirms the complete trajectory of the designed Pappus spiral path when decreasing (red line) and increasing (blue line) radius as shown in Fig. 6f. The *z* coordinate of the unknown focal point is measured with a motorized translation stage with two reference points. The predicted and measured values are shown in Fig. 6g. The experimental values of optical distance lie very close to the predicated values with an average deviation error of 0.12, 0.1, and 0.74 µm in *x*, *y*, and *z* directions, respectively. An increase in the value of error in *z* direction compared to other directions is due to the resolution limit of the motorized stage. Similarly, if the optical distance of an arbitrary focal point with maximum intensity is given, the wavelength of the unknown incident light beam can be accurately measured. The proposed metalens can distinguish small variations in the incident wavelength up to 2 nm. The details are discussed in Supplementary Section 14. The combination of wavelengths and polarization states on focal points offers new tools for characterization, measurement, and detection. The involved polarization state in the super metalenses is linear polarization, which can be extended to include other polarization states, such as circular and elliptical polarization states. The developed super metalenses feature design flexibility, portability, and versatility, which can align well with the continuing trend of device miniaturization and system integration.

## Conclusion

In conclusion, we have designed and developed metasurface-enabled super metalenses for multifaceted control of focal points in 3D space. Geometric metasurfaces are used to realize simultaneous control of phase, polarization, and wavelength of the designed super metalenses. The efficacy of this approach is exemplified through the demonstration of engineered focal points along different 3D trajectories, such as the cylindrical helix, the conical spiral, and the Pappus spiral. Furthermore, we showcase the application of the developed super metalenses in optical distance measurement and wavelength detection in 3D space. These metalenses possess advanced capabilities surpassing those of traditional optical lenses, characterized by accurate 3D positioning of focal points, engineered wavelength, and polarization information, which are impossible with their conventional counterparts. The compact design and unique features of the developed super metalenses may find applications in metrology, imaging, detection, and security.

## Materials and methods

The designed super metalens is realized with plasmonic metasurfaces consisting of gold nanorods on an ITO-coated glass substrate. The glass substrate has an area of 1 × 1 cm^2^. The substrate is first cleaned with acetone for 5 min and then rinsed in isopropyl alcohol (IPA) for 5 min. A nitrogen gun is used to dry the substrate. A polymethyl methacrylate (PMMA) 950 is spin-coated on the substrate at 4000 rpm for 1 min to obtain a film with a thickness of 120 nm, which is baked at 190 °C on a hotplate for 3 min. The electron-beam lithography (Raith PIONEER) is used to expose the PMMA resist for nanopatterning. The accelerating voltage, beam current, and aperture size are 30 kV, 11.7 pA, and 7.5 µm, respectively. The selected writing field is 400 × 400 µm^2^. The exposed PMMA film is developed in the mixture of MIBK: IPA (1:3) for 50 s and rinsed in IPA for 1 min. An electron-beam evaporator is used to deposit a gold film with a thickness of 40 nm on the sample. Finally, the gold nanorods are obtained after the lift-off process in acetone for 12 h.

The metasurface samples are characterized with a home-built optical setup, as shown in Fig. 2d. The combination of a linear polarizer (LP_a_) and a quarter-wave plate (QWP_a_) is used to generate circular polarization states. The incident light beam with tunable wavelengths is provided with a supercontinuum laser source (SuperK EXTREME). The inclined angle of the transmission axis of the LP_a_ and that of the fast axis of QWP_a_ are 0° and 45°, respectively. Different focal points are generated at the predesigned locations in 3D space, depending on the incident wavelength. An objective lens with a magnification of 20× is mounted on a 3D translational stage and used to collect the generated intensity profiles at various observation planes. Another pair of quarter-wave plate QWP_b_ and linear polarizer LP_b_ is used to filter out the nonconverted part on the transmission side. To confirm the polarization rotation angles of focal points with respect to the linear polarization direction of the incident light, both QWP_a_ and QWP_b_ are removed, and the transmission axes of LP_a_ and LP_b_ (analyzer) are perpendicular to each other. A CCD camera is used to capture the intensity patterns.

### Supplementary information

Supplementary Information: Multifaceted control of focal points along an arbitrary 3D curved trajectory

## Data Availability

The data that support the findings of this study are available from the corresponding author upon reasonable request.
