# Supplementary material for: Multifaceted control of focal points along an arbitrary 3D curved trajectory
Source: Light Sci Appl. 2024 Sep 2;13:224. doi: 10.1038/s41377-024-01565-4 (PMC11369200; doi:10.1038/s41377-024-01565-4)
Supplement: Supplementary file 1 — Supplementary Information: Multifaceted control of focal points along an arbitrary 3D curved trajectory [file 41377_2024_1565_MOESM1_ESM.docx]

# **Supplementary Information: Multifaceted control of focal points along an arbitrary 3D curved trajectory**

*Muhammad Afnan Ansari^1^, Hammad Ahmed^1^, Yan Li^2^, Guanchao Wang^1,3^, Jemma E Callaghan^1,4^, Ruoxing Wang^5^, James Downing^4^, Xianzhong Chen^1^**

^1^Institute of Photonics and Quantum Sciences, School of Engineering and Physical Sciences, Heriot-Watt University, Edinburgh EH14 4AS, UK.

^2^School of Materials, Zhengzhou University of Aeronautics, Zhengzhou 450015, China.

^3^ School of Physics, Harbin Institute of Technology, Harbin 150001, China.

^4^ STMicroelectronics, 1Tanfield, Inverleith Row, Edinburgh EH3 5DA, UK.

^5^ Department of Mathematics and Physics, North China Electric Power University, Baoding, 071003, China.

*E-mail: [x.chen@hw.ac.uk](mailto:x.chen@hw.ac.uk)

## **Supplementary Section 1: Transmission spectrum of geometric metasurface**

The proposed super metalenses are designed based on geometric metasurfaces, which consist of gold nanorods with spatially variant orientations sitting on a glass substrate as shown in **Figs. S1a and S1b**. The length, width, and height of each nanorod are 200 nm, 80 nm, and 40 nm, respectively. The size of each pixel is 300 nm along both *x* and *y* directions. The simulated and normalized transmission conversion efficiencies are shown in **Fig. S1c**. The efficiencies are simulated using the frequency domain solver of the Computer Simulation Technology (CST) Microwave Studio software^1,2^. The Drude model is used with plasma frequency equal to 1.37 × 10^16^ rad s^-1^ to calculate the permittivity of gold rectangular nanostructures. The value of collision frequency is 1.215 × 10^14^ rad s^-1^. The glass substrate has a refractive index of 1.46. The unit cell boundary conditions are as follows: period boundaries in *x* and *y* directions and open boundary condition in *z* direction. The curves of calculated transmission conversion efficiencies are relatively uniform in the visible domain from 500 nm to 700 nm as shown in **Fig. S1c**. The conversion efficiency of converted part exceeds 8%. Although the transmission efficiency of the converted part (green curve) is low and the transmission efficiency of non-converted part (black curve) is high, the latter can be filtered out with the experimental setup presented in **Fig. 2d**.


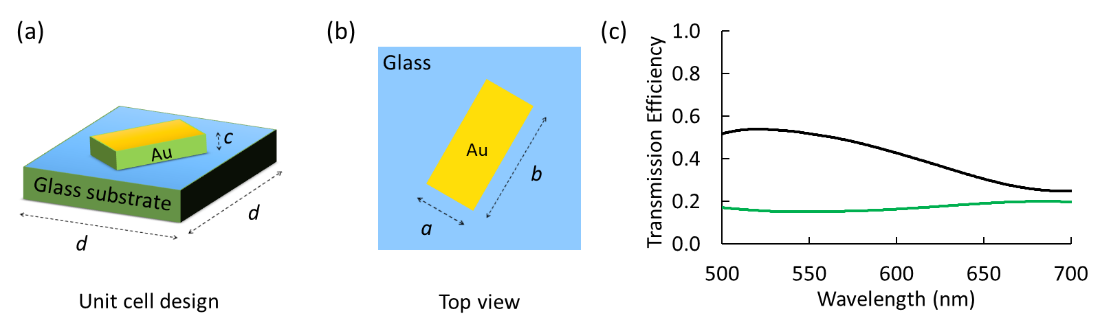


Fig. S1. Transmission spectrum of geometric metasurface. (a) Unit cell design. (b) Top view of unit cell. (c) Simulated converted (green curve) and non-converted (black curve) parts of the transmittance spectra in the visible domain.

## **Supplementary Section 2: Complete intensity distributions and polarization rotation angles of super metalens with 12 focal points**

**Fig. S2** presents simulated and experimental intensity distributions of the proposed super metalens M_1_ with 12 focal points on 3D cylindrical helix path. There are 12 wavelengths and 12 unique linear polarization rotation angles. The wavelengths and longitudinal distance vary from 480 nm to 700 nm and 300 µm to 520 µm, respectively. Both the step size of the wavelength and that of longitudinal distance are 20-units. Intensity distributions in first and second rows are obtained under the illumination of RCP incident light beams. The intensity distributions in each third row are obtained under the illumination of LP incident light beams with an analyzer having the direction perpendicular to the incident polarization direction. The dark intensity patterns confirm the presence of designed polarization rotation angles at each focal point. The dotted box shows the region of corresponding focal spot with solid white arrow which depict the initial polarization rotation angles. The dashed white and solid yellow arrows represent the direction of incident light’s polarization and the transmission axis of the analyzer, respectively.


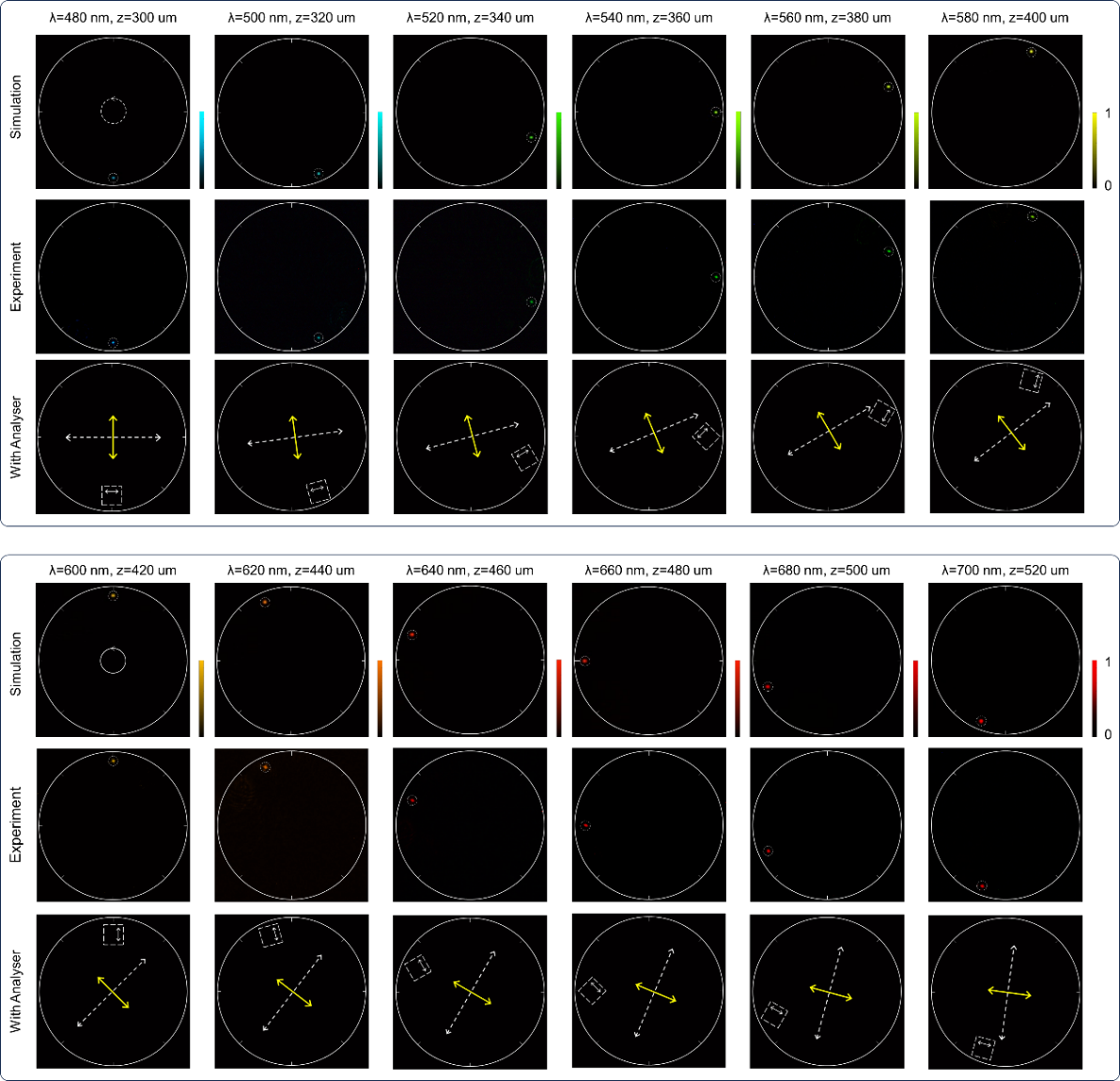


Fig. S2. Super metalens with 12 focal points along a cylindrical helix trajectory. The polarization state of the incident light for the first two rows and that for the third rows are right circular polarization and linear polarization, respectively. The size of each figure is 70 µm along both *x* and *y* directions.

**Supplementary Section 3: Analysis of the focal point, accuracy, spot profile, and FWHM**

The intensity distributions of focal points generated by super metalens M_2_ at different wavelengths and at their corresponding focal regions along the *z*-direction are presented in **Fig. S3a**. In order to compare the details of focal spots at different 3D observation positions, the range in the xy plane and that along the light propagation that we use are same. It is worth mentioning that these focal spots are not along the optic axis. *r* represents the off-axis direction on the *x*y plane. On the corresponding trajectory of super metalens M_2_, all focal points have a fixed off-axis distance of 30 μm. The focal distance (the location of maximum intensity) is increased by 0.5 μm when the incident wavelength and the designed focal distance are 500 nm and 300 μm, respectively. For other focal points, the calculated focal deviation increases by 0.1 μm for every 100 μm increase in the designed focal distance. In the off-axis direction on the *xy* plane, as the focal length increases, the off-axis angle decreases, resulting in a decrease in the focal deviation. At the incident wavelength *λ* = 500 nm and the designed focal distance *f* = 300 μm, the deviation is maximum in the off-axis radial direction, which is 21 nm.

The intensity distributions of focal points at different wavelengths and designed focal planes are also presented in *rr_Ʇ_* plane (**Fig. S3b**) with their FWHM values and focal deviations *dr* in both *r_Ʇ_*-direction (**Fig. S3c**) and *r*-direction (**Fig. S3d**). Where, *r_Ʇ_* represents the direction perpendicular to the off-axis direction of the focal point on the *xy* plane. For example, if a focal point at the wavelength of 500 nm is designed to focus on the *xy* plane with coordinates (0 μm, -30 μm), then *r* will represent the negative *y*-direction and *r_Ʇ_* will represent the *x*-direction. Due to the influence of intrinsic dispersion, the focal spot profiles changes as the focal length increases. These changes mainly occur in the *r_Ʇ_*-direction compared to the *r*-direction. The change in focal deviations *dr* and FWHM values are less in the *r*-direction compared to the *r_Ʇ_*-direction. The maximum focal deviation *dr* in *r*-direction is less than 60 nm.


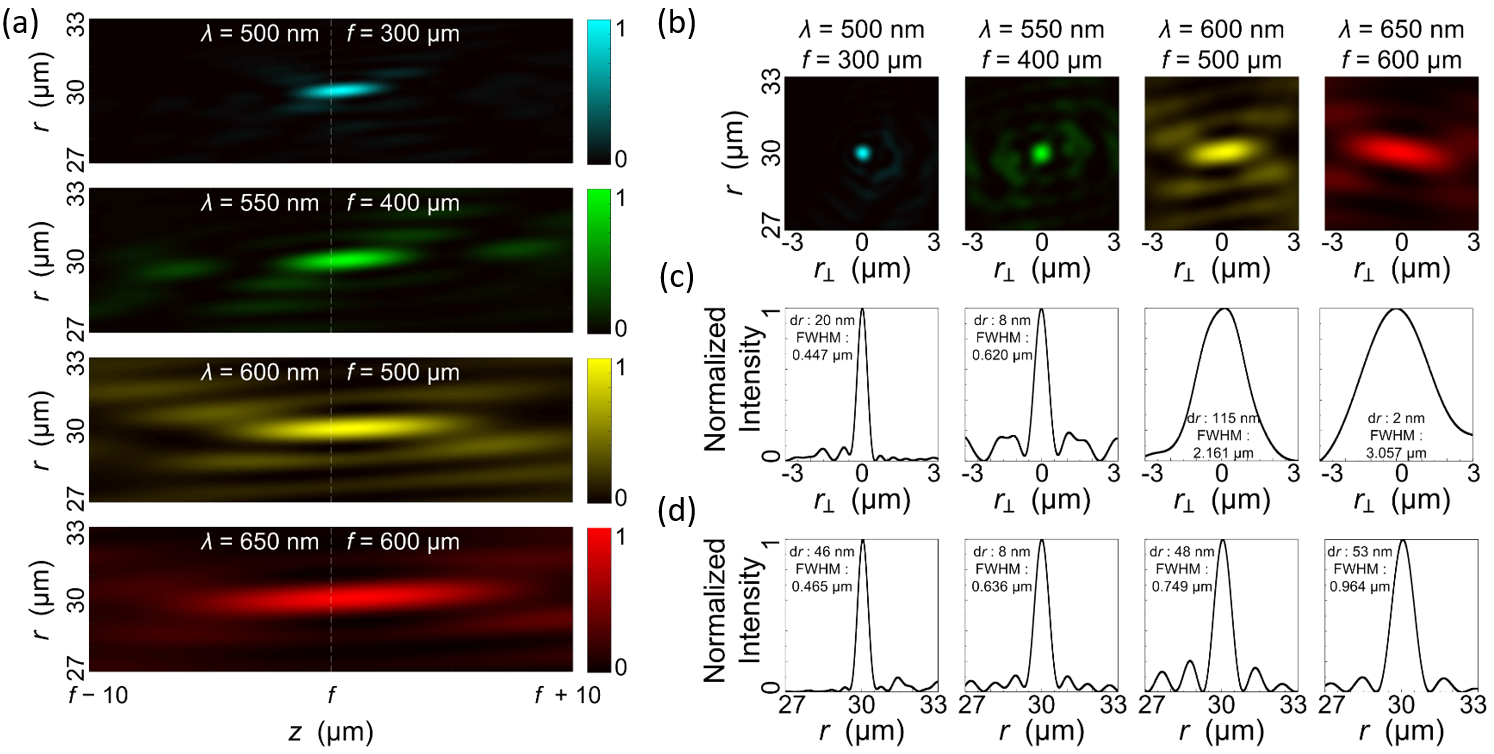


Fig. S3. Analysis of the focal point, accuracy, spot profile, and FWHM. (a) The intensity distributions of focal points at different wavelengths on their respective focal regions in the (a) *rz* plane, (b) *rr_Ʇ_* plane, (c) *r_Ʇ_*-direction, and (d) *r*-direction.

**Supplementary Section 4. Effect of aperture size on the focal spot**

The size and diffraction of a focal spot are influenced by the focal length *f* and metalens aperture size *S*. **Fig. S4a** presents the states of a single focal point generated by the metalenses with different focal lengths *f* and different metalens aperture sizes *S*. The selected metalens aperture sizes are *S*_1_ = 300×300 μm^2^, *S*_2_ = 350×350 μm^2^, *S*_3_ = 400×400 μm^2^ and, *S*_4_ = 450×450 μm^2^. It can be seen in **Fig. S4a** that when the metalens aperture size remains unchanged, the size of the focal spot increases with the increase in focal length of the metalens. In contrast, for a fixed focal length, the size of the focal spot decreases as the aperture size of the metalens increases. The relationships between the normalized focal spot diameter *d* / *λ* (*d* is the diameter of the focal spot) and the focal length *f* with different metalens aperture sizes *S* are presented in **Fig. S4b**. The normalized focal spot diameter agrees with the expression *d* / *λ* = 2*f* / *D*, where *D* is the clear aperture of the metalens. In addition, the smaller size of the focal spot will have larger intensity of the diffraction ring formed by higher-order diffraction. It can be seen in **Fig. S4c** that when the focal length is 300 μm, a metalens with a larger aperture size *S*_4_ has a smaller focal spot diameter and a stronger first-order diffraction ring.


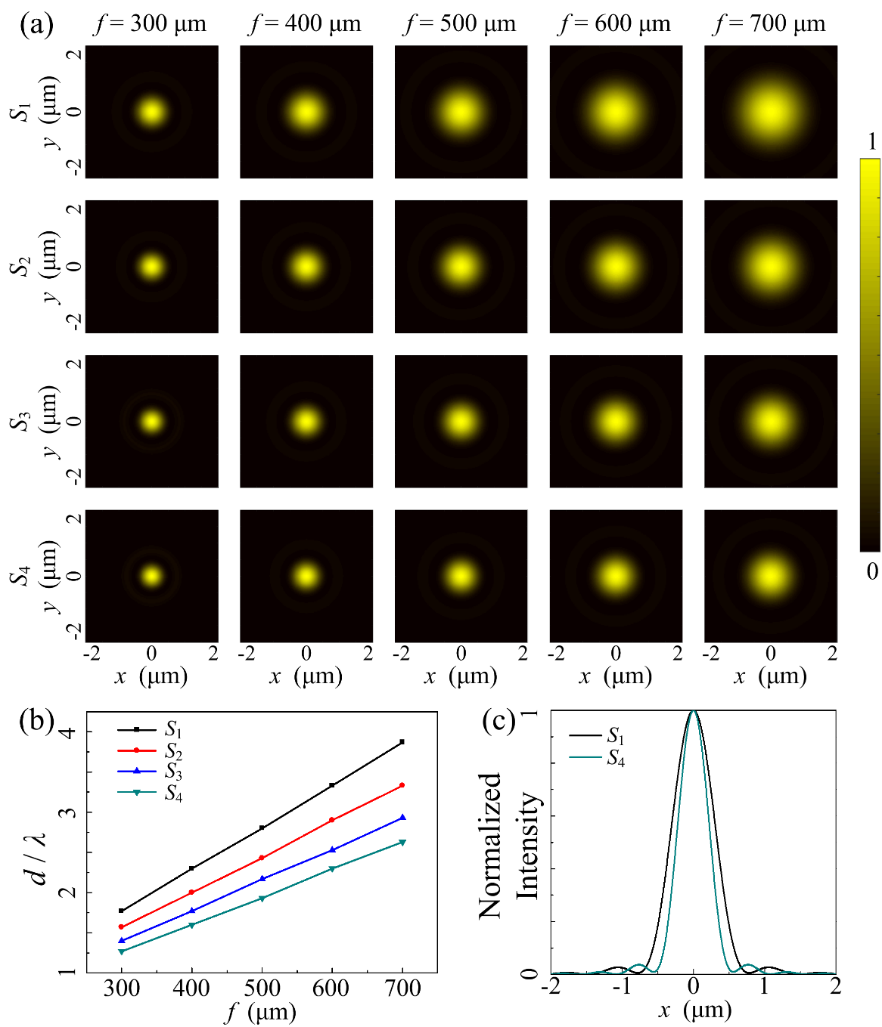


**Fig. S4. Effect of aperture size on the focal spot.** (a) Normalized intensity distributions at the focal plane of the metalenses with different combinations of focal lengths *f* and metalens aperture sizes *S*. (b) Relationship between the normalized focal spot diameter of the single focal point and the focal length with the metalens aperture sizes *S*_1_ = 300×300 μm^2^ (black line), *S*_2_ = 350×350 μm^2^ (red line), *S*_3_ = 400×400 μm^2^ (blue line), and *S*_4_ = 450×450 μm^2^ (green line). (c) Intensity profile of a single focal spot (*f* = 300 μm) in the radial direction with the different metalens aperture sizes *S*_1_ (black line) and *S*_4_ (green line).

**Supplementary Section 5. Effect of the longitudinal distance and longitudinal separation on the focal spot**

Here, the super metalens M_2_ with *Δz* = 1 µm is chosen for the first case. First two rows in **Fig. S5** show the simulation and experimental results under the illumination of RCP incident light beam. It is noted that the size of corresponding focal point is not significantly increased with the change of longitudinal distance *z* for the designed super metalens with longitudinal separation *Δz* = 1 µm. Therefore, longitudinal separation of *Δz* = 1 µm can be used in the design. The 2^nd^ case of *Δz* = 2 µm is discussed in **Fig. 3**. The dark intensity regions in third row confirms the predesigned polarization state under the illumination of LP incident light beam with an analyzer at the output as shown in **Fig. S5**. The values given next the dotted yellow circles are the initial polarization rotation angles and are confirmed using Malus’ law.


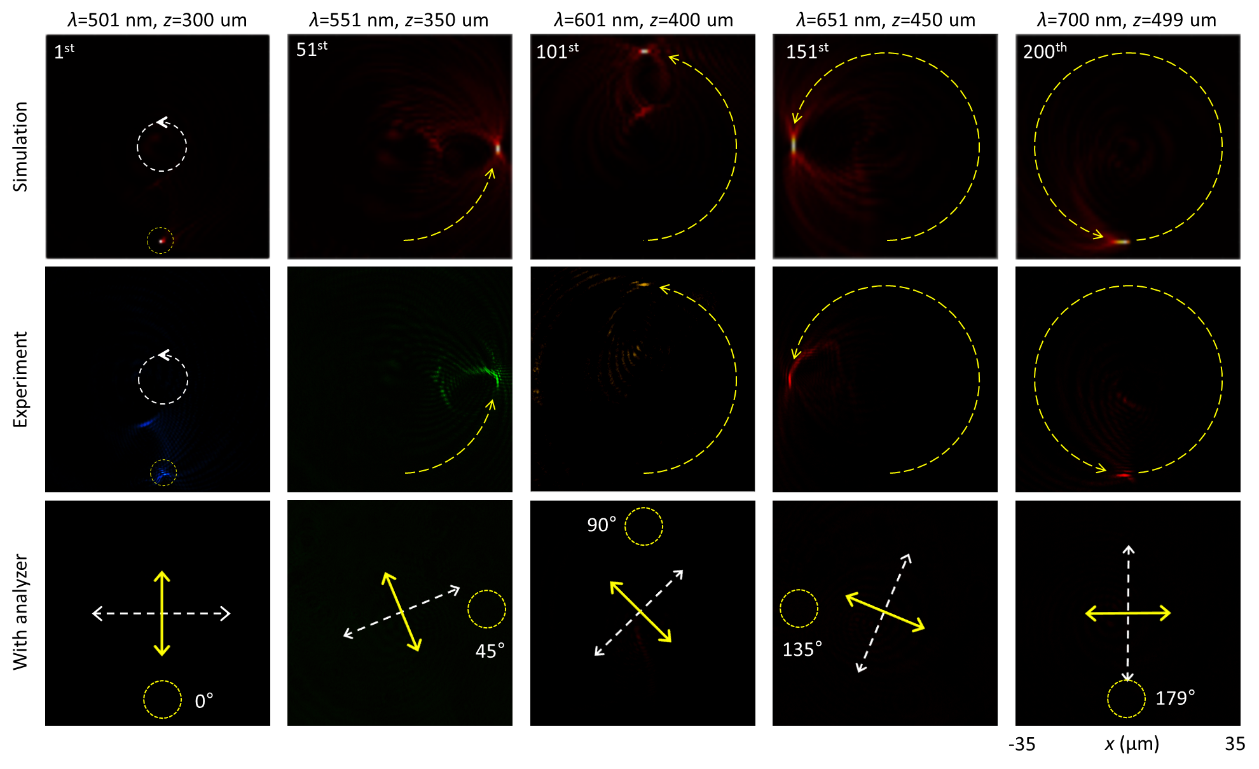


Fig. S5. Super metalens with a longitudinal separation *Δz* = 1 µm. The dashed yellow curves are drawn as a reference of 3D optical trajectory. Solid yellow and dashed white arrows represent the transmission axis of the analyzer and incident polarization direction, respectively.

The super metalens M_2_ with *Δz* = 5 µm is chosen for the third case. **Fig. S6a** presents the schematic of the super metalens with total optical length T_L_ = 995 µm and *Δz* = 5 µm. Therefore, the initial and final values of the longitudinal distances are 300 µm and 1295 µm, respectively. The intensity distributions of the focal points under the illumination of RCP incident light beams are shown in **Fig. S6b**. It is noted that the size of corresponding focal point is notably increased with the change of longitudinal distance *z* for the designed super metalens with longitudinal separation *Δz* = 5 µm. Therefore, according to **Fig. S5** (*Δz* = 1 µm), **Fig. 3** (*Δz* = 2 µm), and **Fig. S6** (*Δz* = 5 µm), smaller values of *Δz* generate negligible or less effect on the size of focal spot when the longitudinal distance is changed.


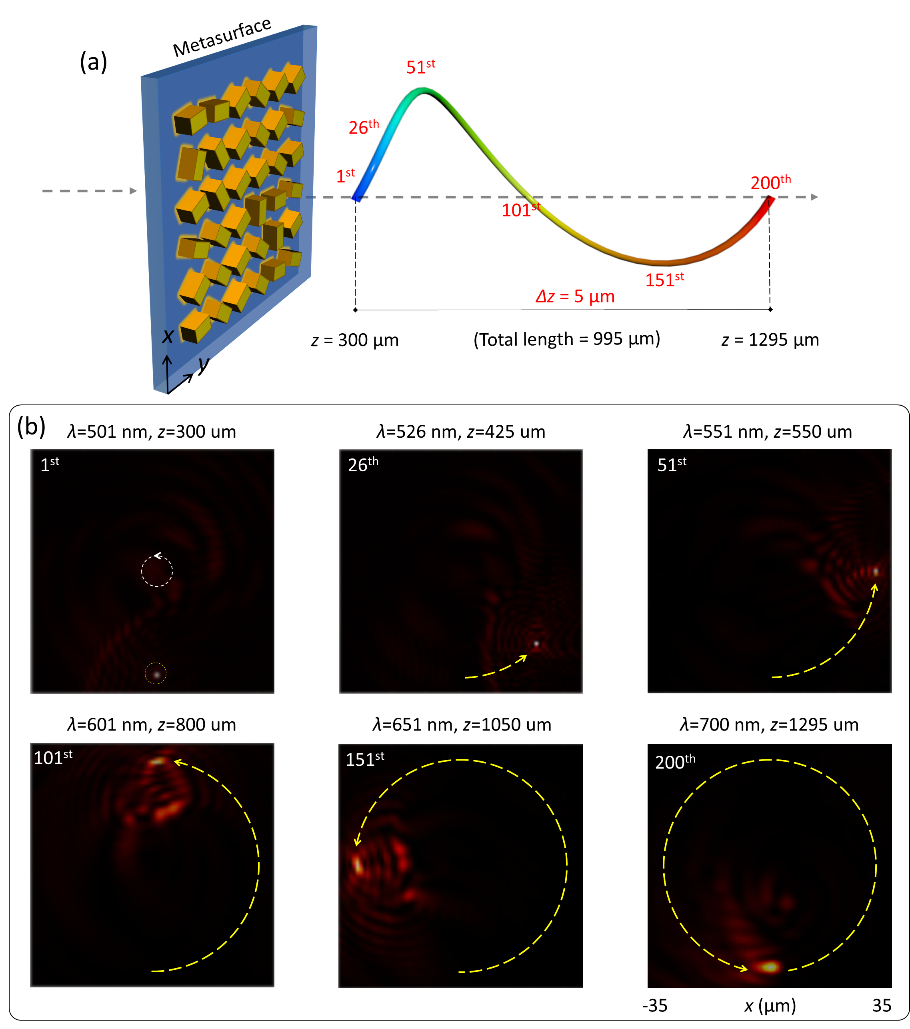


Fig. S6. Super metalens with a longitudinal separation *Δz* = 5 µm. (a) The schematic diagram of super metalens M_2_ with continuous single-cycle 3D cylindrical helix path and with *Δz* = 5 µm. The total length of cylindrical helix is equal to 995 µm with initial and final values of 300 µm and 1295 µm, respectively. (b) Intensity distributions of selected focal points (1^st^, 26^th^, 51^st^, 101^st^,151^st^, and 200^th^) on a single-cycle 3D cylindrical helix trajectory at corresponding operating wavelengths (501 nm, 526 nm, 551 nm, 601 nm, 651 nm, and 700 nm) and longitudinal distances (300 µm, 425 µm, 550 µm, 800 µm, 1050 µm, and 1295 µm, respectively). The results are obtained under the illumination of RCP incident light beam. Dashed yellow curves with arrows represent the trajectory of single-cycle 3D cylindrical helix with constant radius.

## **Supplementary Section 6: Analysis of focal points in different observation planes**

**Fig. S7** presents a comprehensive analysis of focal points with the cross-sectional distribution along the *z*-axis. Upon the illumination of a laser beam at 601 nm, a sequence of intensity distributions across various observation planes are given in **Fig.S7a**. The predesigned central observation plane is located at *z* = 502 µm. We can clearly see the results at different planes, i.e., *xy* (**Figs. S7a** and **S7b**), *yz*, and *xz* planes (**Fig. S7c**). The intensity distribution results in all the planes clearly show the existence of the focal point with the highest intensity at the designed position (*x* = 0 µm, *y* = 30 µm, and *z* = 502 µm). The undesired low intensity noise appears due to the intrinsic dispersion of adjacent focal points, which is discussed in **Supplementary Section 8** and **9**.


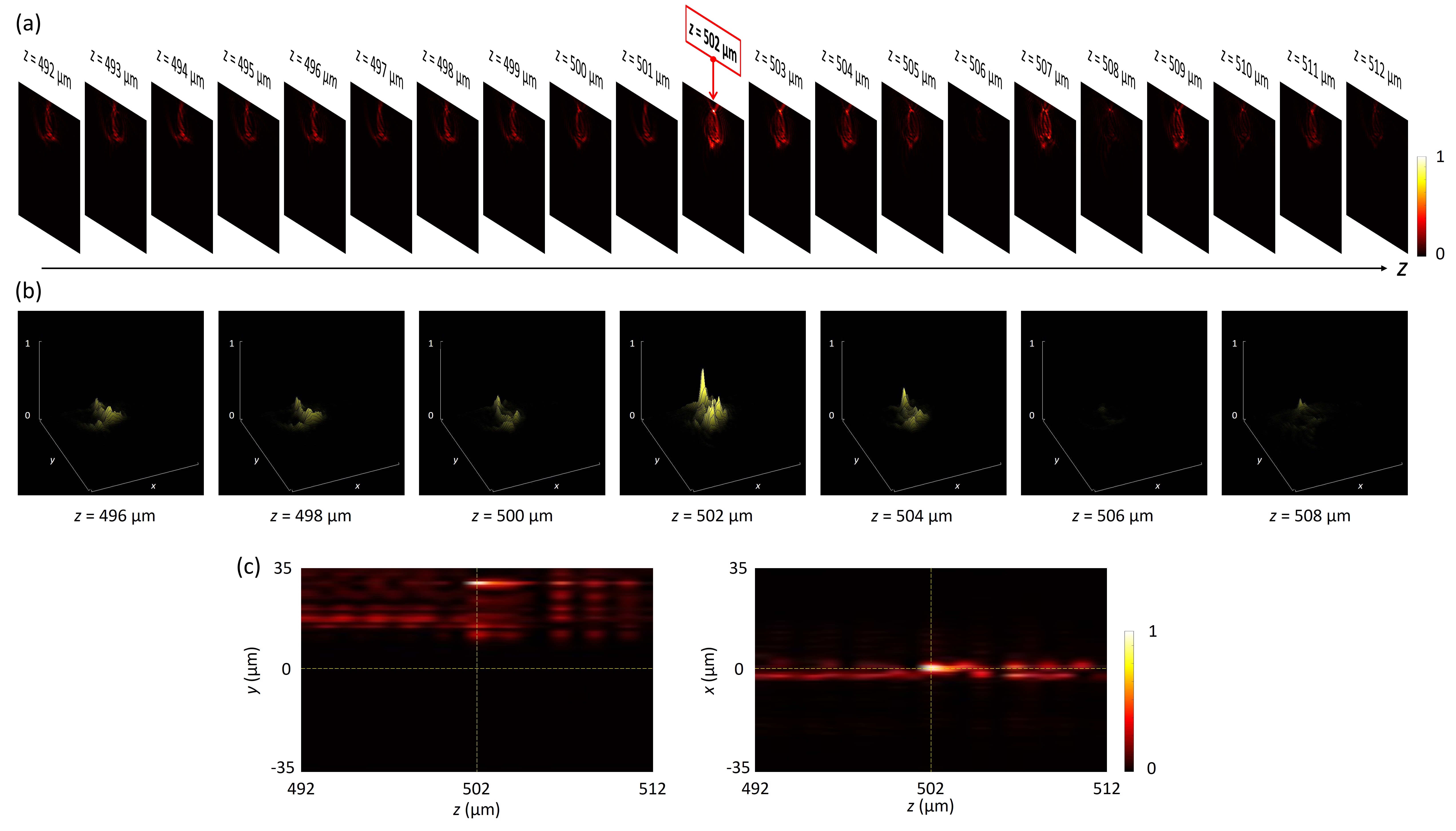


Fig. S7. Analysis of focal point in different observation planes.

## **Supplementary Section 7: Focusing efficiency of the super metalens**

The focusing efficiency of a metalens is defined as the ratio of the intensity of electric field in a circular aperture with 3× FWHM of the focal point to the total intensity of the incident light beam^3–5^. We calculate the focusing efficiency of metalens at different wavelengths and different focal planes based on the single-cycle 3D cylindrical helix trajectory. The mesh size is 0.25 µm along both *x* and *y* directions. The focusing efficiency *η_f_* of the proposed super metalens with 200 focal points located along a single-cycle 3D cylindrical helix varies from 0.82% to 0.43% as the wavelength varies from 500 nm to 700 nm, respectively, as shown in **Fig. S8**.


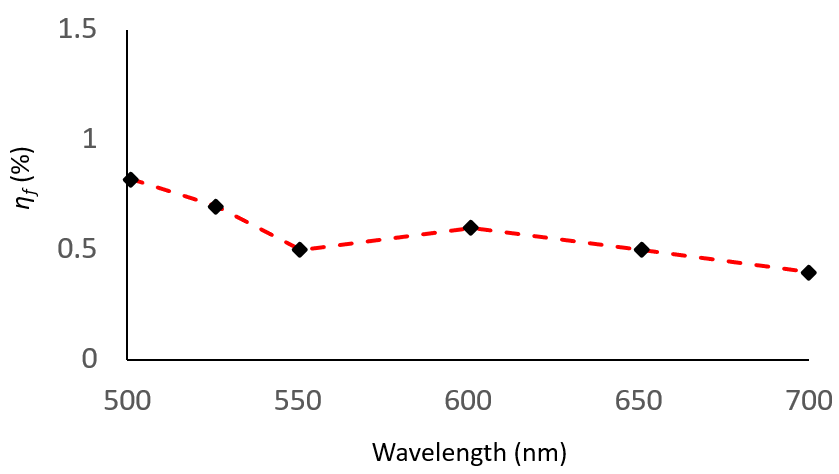


Fig. S8. Focusing efficiency *η_f_* of the super metalens.

## **Supplementary Section 8: Effect of intrinsic dispersion on other focal positions**

The super metalens M_1_ (with 12 focal points) is chosen to illustrate the effect of intrinsic dispersion of wavelengths at other focal positions. **Figs. S9a** and **S9c** present the corresponding predesigned focal points at the desired focal planes *z* = 500 µm and *z* = 520 µm at *λ* = 680 nm and *λ* = 700 nm, respectively. To see the effect of intrinsic dispersion, intensity distribution is obtained at a focal plane other than the designed focal plane (*z* = 510 µm) at a higher-designed wavelength (*λ* = 700 nm). The behaviour (depicted in **Fig. S9b**) exists due to the relatively small intrinsic dispersion. For the focal point of a smaller-designed wavelength, the light beam is converged before 510 µm focal plane. In contrast, for a focal point of a higher-designed wavelength, the light beam is converged after 510 µm focal plane.


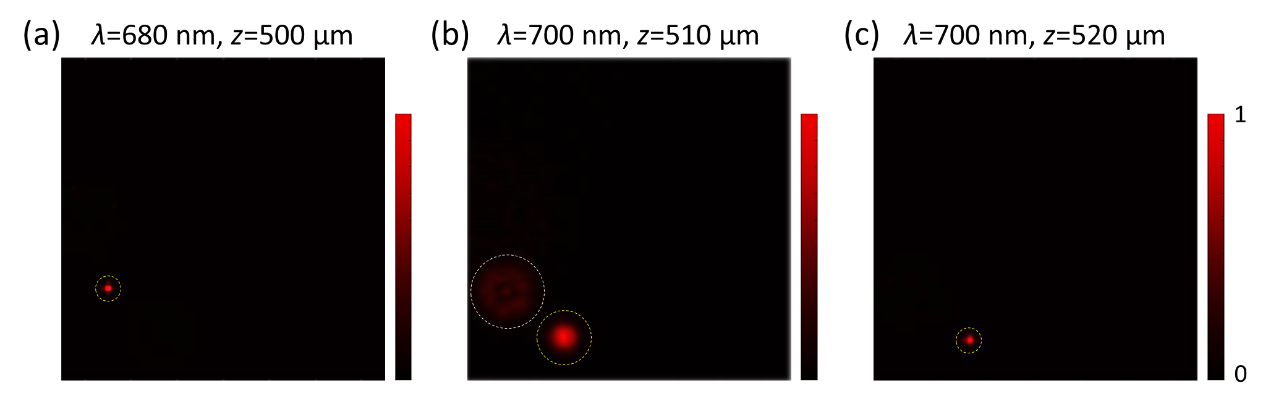


Fig. S9. Effect of dispersion of the focusing condition at focal planes other than the designed focal planes. Super metalens M_1_ with 12 focal points at (a) smaller-designed wavelength and at a designed focal plane, (b) higher-designed wavelength and at a focal plane other than the designed focal planes, and (c) higher-designed wavelength and at the designed focal plane.

## **Supplementary Section 9: Effect of number of focal points (*N*) and longitudinal separation (*Δz*) on the level of crosstalk**

A crosstalk can arise among focal points that lie very close to each other due to the small wavelength difference and intrinsic dispersion. However, the intrinsic dispersion is not enough to completely converge one boundary wavelength at the other boundary wavelength focal position due to the selection of predesigned curved trajectories. Each focal position has unique *x*, *y* and *z* coordinates which do not overlap in 3D space. As a result, the undesired focal points with low intensities appear alongside the desired high intensity focal point. Therefore, we utilize a method to observe only the highest intensity focal point to identify the modulated focal position by the proposed super metalens. There are four factors that can affect the level of crosstalk, i.e., longitudinal separation (*Δz*) and number of focal points (*N*), the selection of 3D trajectory, and bandwidth of light source. The higher number of focal points *N* and the lower values of *Δz* can increase the level of crosstalk as illustrated in **Fig. S10**. It is also noted that the bandwidth of supercontinuum laser source is slightly higher for longer wavelengths compared to the shorter wavelengths as shown in **Fig. S11**. Therefore, due to the bandwidth of light beam at a specific wavelength (several nanometers), some unwanted focal points also appear alongside the desired focal point at certain positions for example at 51^st^, 101^st^, and 151^st^ in **Fig. 3**.


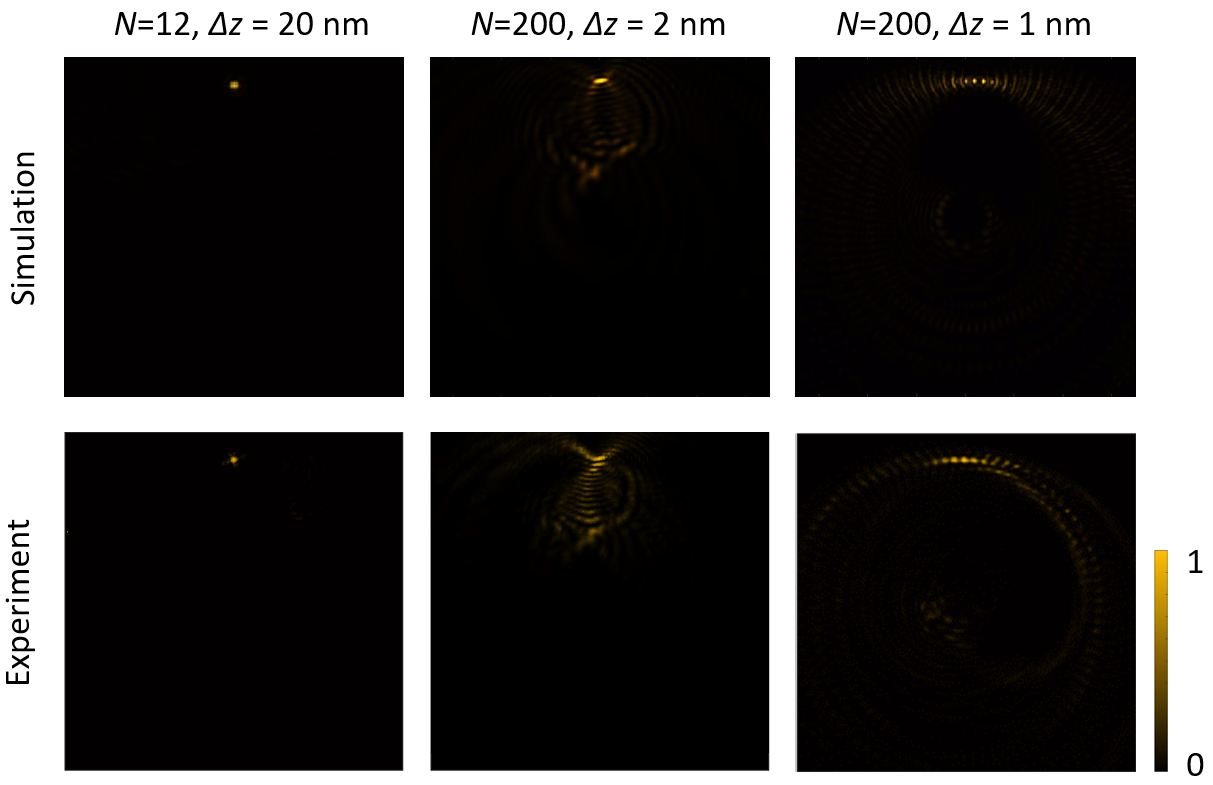


Fig. S10. Effect of number of focal points (N) and longitudinal separation (Δz) on the level of crosstalk.


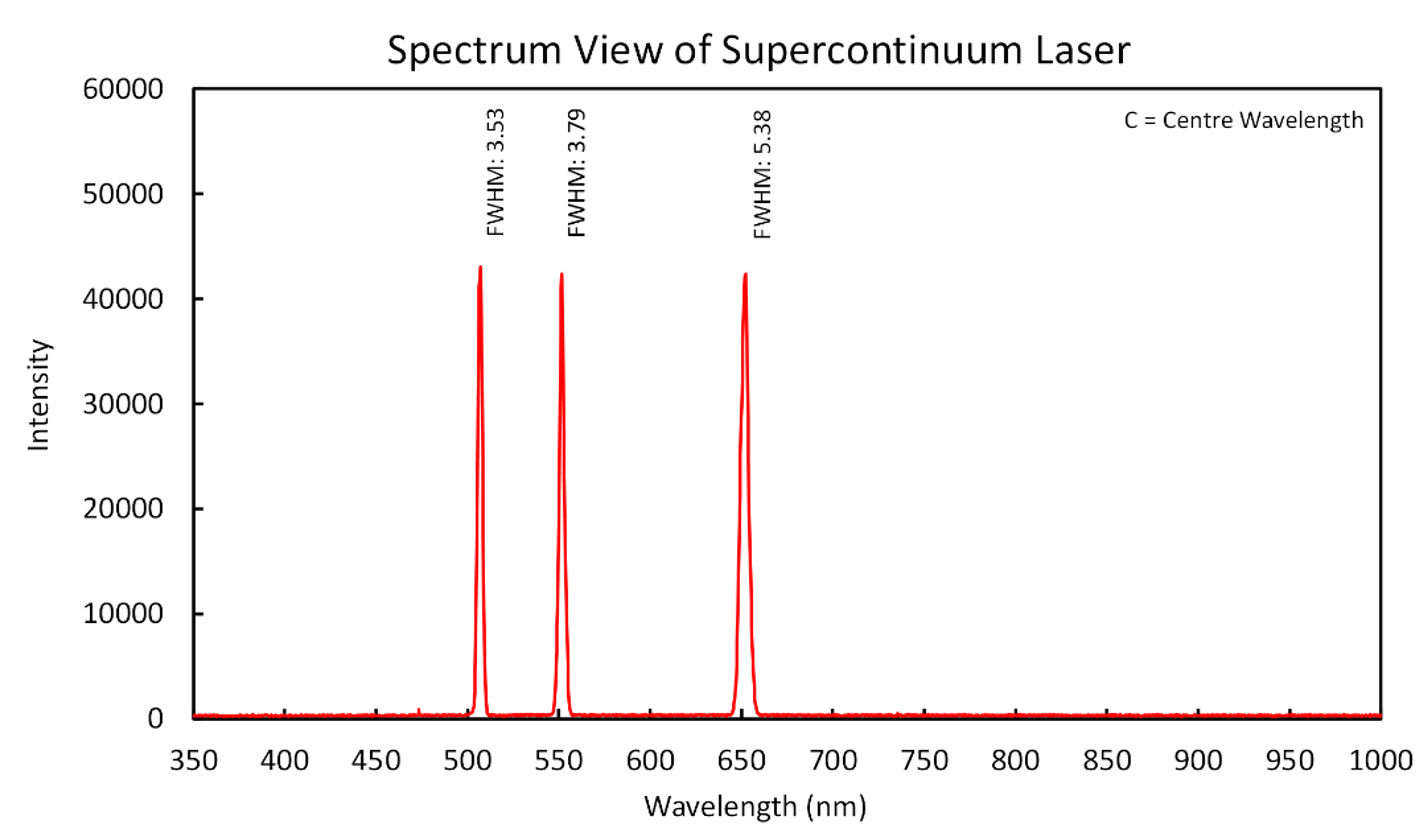


Fig. S11. Wavelength bandwidth of the supercontinuum laser at specific wavelengths measured by a commercial high precision spectrometer.

**Supplementary Section 10. Super metalens with focal points along a continuous 3D conical spiral with an increasing radius**

The design M_4_’ is inspired by the 3D conical spiral structure with increasing radius along the longitudinal direction as shown in **Figs. S12a** and **S12b**. The curved optical path of a conical spiral is governed by the parametric **Eq. 6** with *C* = 2 and variable radius *R* = *r_n_*. Where $r_{n}=R_{i}+(n-1)\Delta R$ is an increasing function with initial value of radius *R_i_* and incremental shift *ΔR*. The initial value of radius is 0 and final value of radius is 30 µm. **Fig. S12c** shows the simulation results of super metalens M_4_’ with focal points along 3D optical curved trajectory governed by the conical spiral shape at corresponding wavelengths and longitudinal distances. For an illustration purpose, three points on the optical path are chosen to prove the concept i.e., 1^st^, 100^th^, and 200^th^. The value of radius at 1^st^ focal point should be the initial radius value (0), which is clearly evident due the presence of a focal point with highest intensity at the centre at 501 nm wavelength. According to the design M_4_’, the value of radius should become exactly half at the 600 nm wavelength and hence, the highest intensity focal point is appeared in the middle of the conical spiral trajectory. At the end of the conical spiral (*z* = 499 µm), the focal point with highest intensity appears at the position where the radius reaches the maximum (30 µm) at 700 nm.


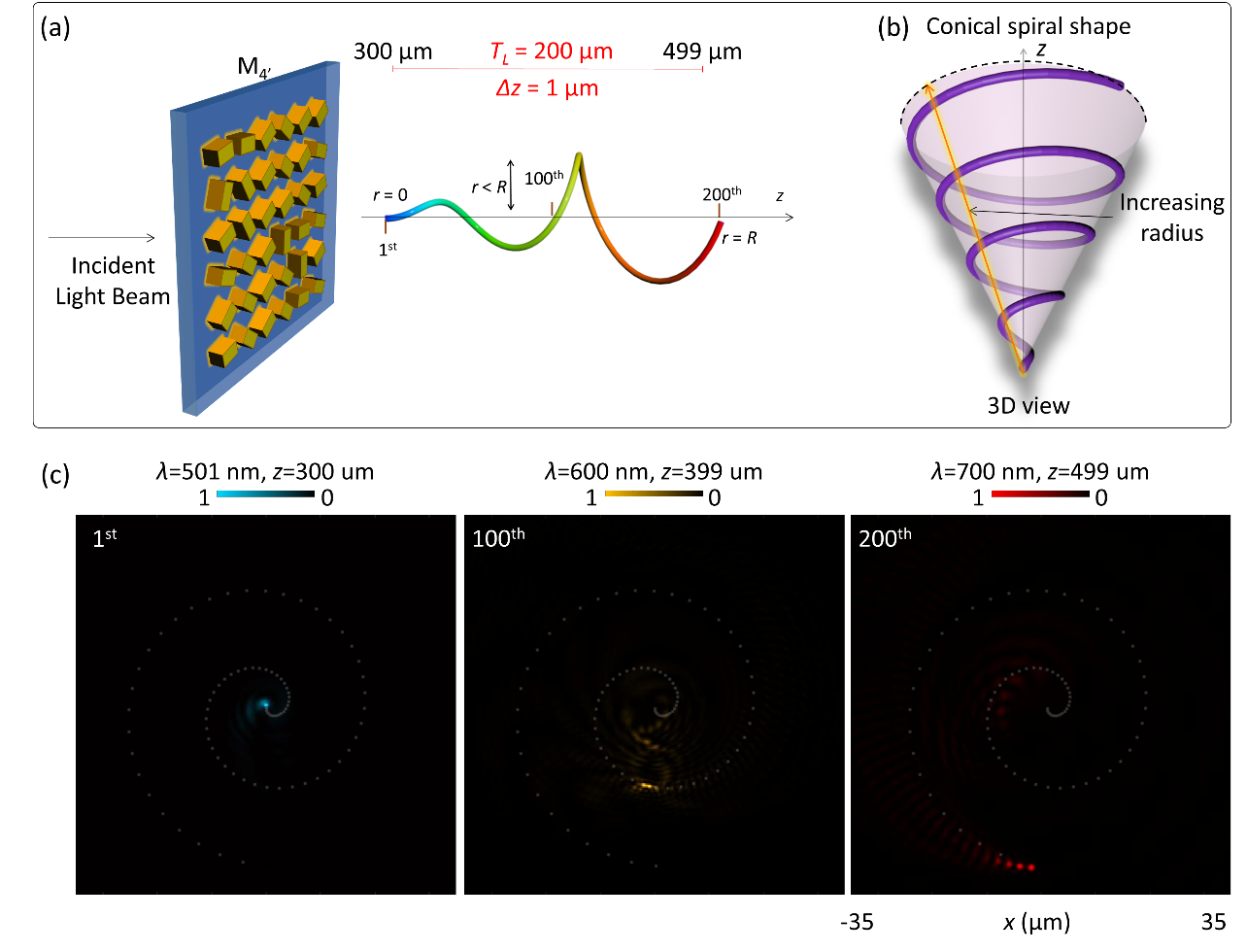


Fig. S12. Super metalens with focal points along a continuous 3D conical spiral with an increasing radius. (a) The schematic diagram of super metalens M_4_’ generating 200 focal points along a continuous 3D conical spiral optical trajectory with increasing radius. The minimum value of radius is 0 and maximum value is 30 µm. The wavelength is modulated with operating range from 501 nm to 700 nm with step size of 1 nm. (b) The 3D view of conical spiral shape with an increasing radius. (c) The simulated intensity distributions of designed super metalens for selected focal points on the 3D conical spiral trajectory under the illumination of RCP incident light beam. The results for selected focal positions (1^st^, 100^th^, and 200^th^) are acquired at the operating wavelengths (501 nm, 600 nm, and 700 nm) and longitudinal distances (300 µm, 399 µm, and 499 µm, respectively). The dotted white spiral curves are drawn to illustrate the conical spiral trajectory with increasing radius in 3D space.

## **Supplementary Section 11: Polarization rotation angles along the Pappus spiral**

In **Fig. S13**, first rows represent the intensity profiles and the presence of focal points under the illumination of RCP light beam. The dark intensity regions are shown in the last row which are the experimental results with analyzer under the illumination of LP incident light beam. The results confirm the presence of the unique predesigned linear polarization rotation angles. The values written next to the dashed yellow circles depict the initial polarization rotation angles of each point when *β* = 0°.


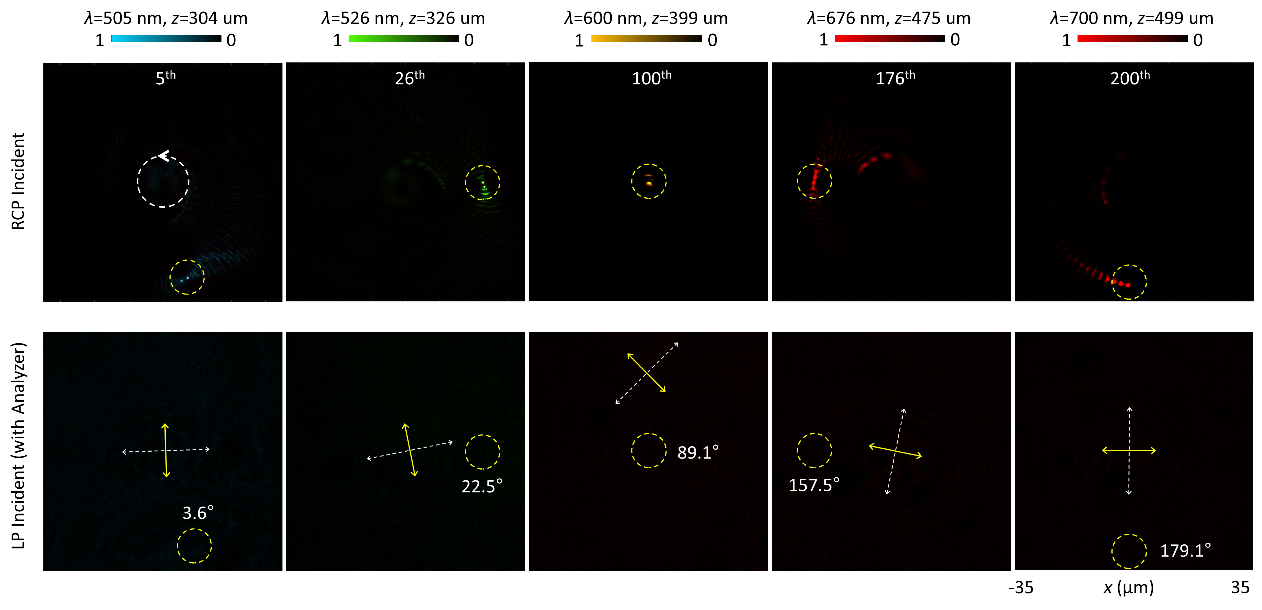


Fig. S13. Confirmation of linear polarization rotation angles along the Pappus spiral trajectory. First row shows the intensity distributions of the selected focal points (5^th^, 26^th^, 100^th^,176^th^, and 200^th^) along Pappus spiral trajectory at corresponding operating wavelengths (505 nm, 526 nm, 600 nm, 676 nm, and 700 nm) and longitudinal distances (304 µm, 326 µm, 399 µm, 475 µm, and 499 µm, respectively). First row contains simulation results under the illumination of RCP incident light beam. The 2^nd^ row presents the intensity distributions for the confirmation of designed polarization rotation angles under the illumination of LP incident light beam with an analyzer at the output. The direction of analyzer (solid yellow arrow) is always perpendicular to the polarization direction of incident light beam (dashed white arrow). The regions of corresponding focal points are highlighted with dashed yellow circles.

## **Supplementary Section 12: Effect of different 3D curved trajectories on the performance of the super metalens**

Different 3D curved trajectories are used in this work i.e., single, and double cycle cylindrical helix, conical, and Pappus spirals. To observe the effect of different shapes of 3D trajectories on the performance of the proposed super metalens, the intensity distributions of focal points of different 3D curved shapes with different unwanted noise are presented in **Fig. S14**. To analyse the effect of unwanted noise on the metalens performance, intensity distributions of the 200^th^ focal point at 700 nm are simulated and measured. It is noted that different 3D curved trajectories have different levels of noise under the same condition which affects the performance of the metasurface. For example, the Pappus spiral curved trajectory is a more complex curved trajectory than single or double helix, therefore possessing higher level of unwanted noise. The greater number of focal points and smaller neighbouring distance between the adjacent focal points in *x*, *y* and *z* directions can contribute toward the complexity of 3D curved trajectory with higher intrinsic dispersion. Therefore, the 3D trajectories and shapes that require a greater number of focal points *N* with smaller values of *Δz* will have a higher level of noise as shown in **Fig. S10**. The effect of unwanted noise due to high intrinsic dispersion can be minimized by optimizing the number of focal points *N* and the neighboring distance between the adjacent focal points.


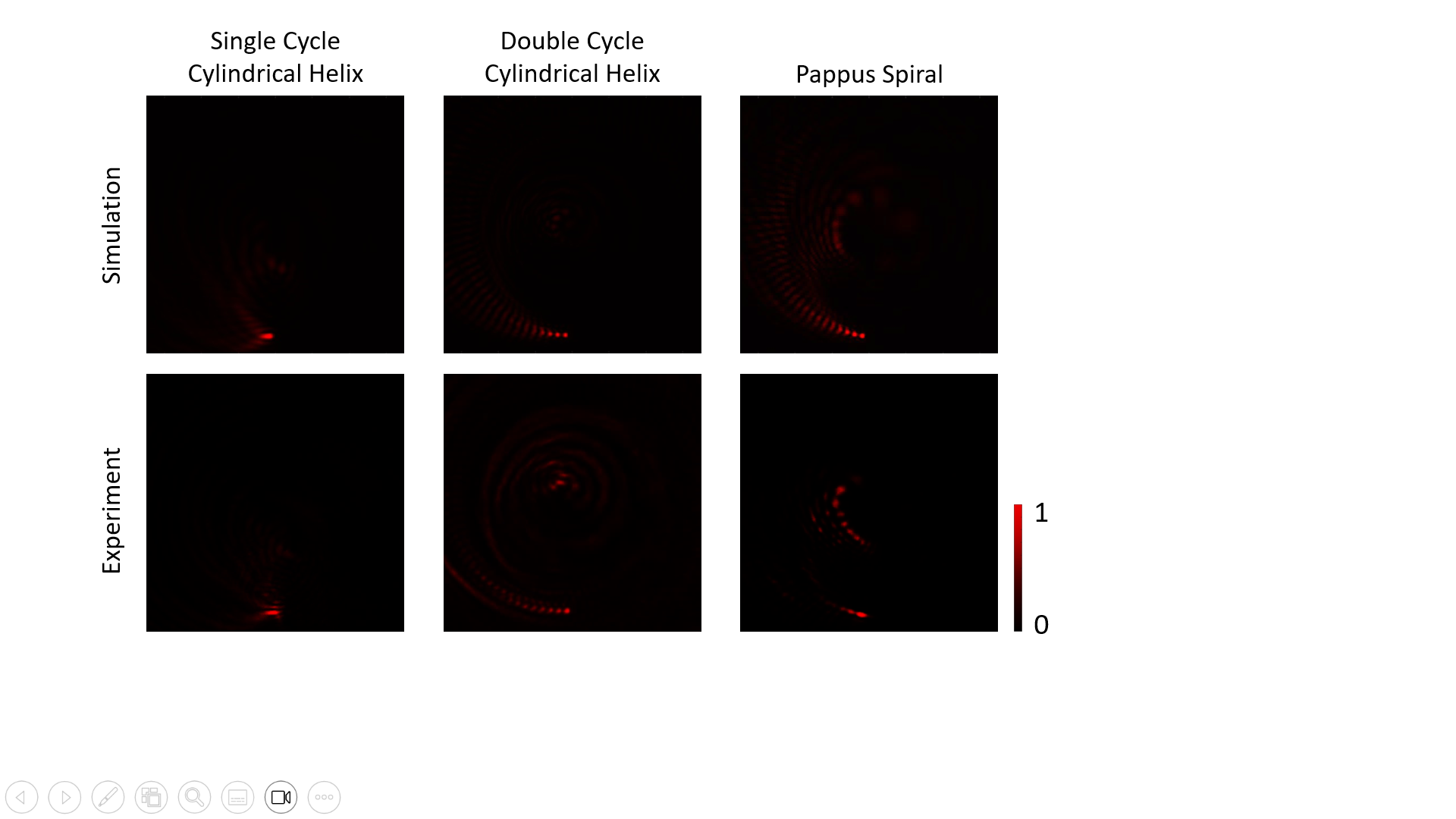


Fig. S14. Effect of 3D curved trajectories on the performance of the super metalens.

## **Supplementary Section 13: Comparison of proposed metalens with other metalenses in our previous works**

In this work, we propose and experimentally demonstrate the multifaceted control of focal points along the customized 3D arbitrary curved trajectories and its application in 3D optical distance measurement. The wavelengths and polarization states of all focal points are engineered in a desirable manner. The novel metalens can simultaneously realize customized 3D positioning, polarization states, and wavelengths of focal points. Unlike previous works, this unique functionality is achieved by using more control variables, including compression (*N*, *Δz*), wavelength (*λ*), longitudinal distance (*z*), polarization rotation angle (*Γ*) and position coordinates (*x*, *y*). Benefiting from more design flexibility, the metalens possesses unique features, which are different from those in previously reported works^1,2,6,7^. These advanced capabilities surpass those of traditional optical lenses, characterized by accurate 3D positioning of focal points, engineered wavelength and polarization information, which are impossible with their conventional counterparts. **Table S1** compares this work and previous works in terms of design, functionality, and applications.

| Work | Functionality | Operating wavelengths | Multidimensional space control | Longitudinalcontrol | Polarization control | Applications |
| --- | --- | --- | --- | --- | --- | --- |
| [6] | Creating 2D polarization structures | 650 nm | Fixed 2D and 3D | No | Yes | 2D and 3D polarization structure generation |
| [1] | Creating color-selective 3D knots | 500 nm, 575 nm, 650 nm | Fixed 3D | No | Yes | Generating Multiple 3D polarization knots |
| [2] | Creating multiple 3D knots along light propagation. | 650 nm | Fixed 3D | Yes | Yes | 3D image steganography |
| [7] | Metalens Spectrometer | 500 nm to 679 nm | Only 2D | No | No | Wavelength separation and detection |
| **Our Work** | Simultaneous realization of customized 3D positioning, polarization states, and wavelengths | A linearly polarized incident beam with continuously variant wavelengths. 501 nm to 700 nm (up to 1 nm step size). | Focal spot changes along the arbitrary 3D trajectory by controlling the incident wavelength | Yes | Yes | Optical distance measurement in 3D space |

Table S1. Comparison of proposed metalens with other metalenses in our previous works.

## **Supplementary Section 14: Effect of small variation in the incident wavelength on the focal point**

To observe the effect of subtle variations, the results of intensity distributions at various wavelengths with a very small variation up to 2 nm are provided in **Fig. S15**. It is confirmed that the proposed super metalens can distinguish subtle variations in the incident wavelength up to 2 nm. The focal point with the highest intensity accurately moves to the predesigned location on a 3D curved trajectory of cylindrical helix when the incident wavelength is varied from 550 nm to 560 nm with a step size of 2 nm as shown in **Fig. S15a-f**, respectively. The results are obtained at their corresponding longitudinal distances.


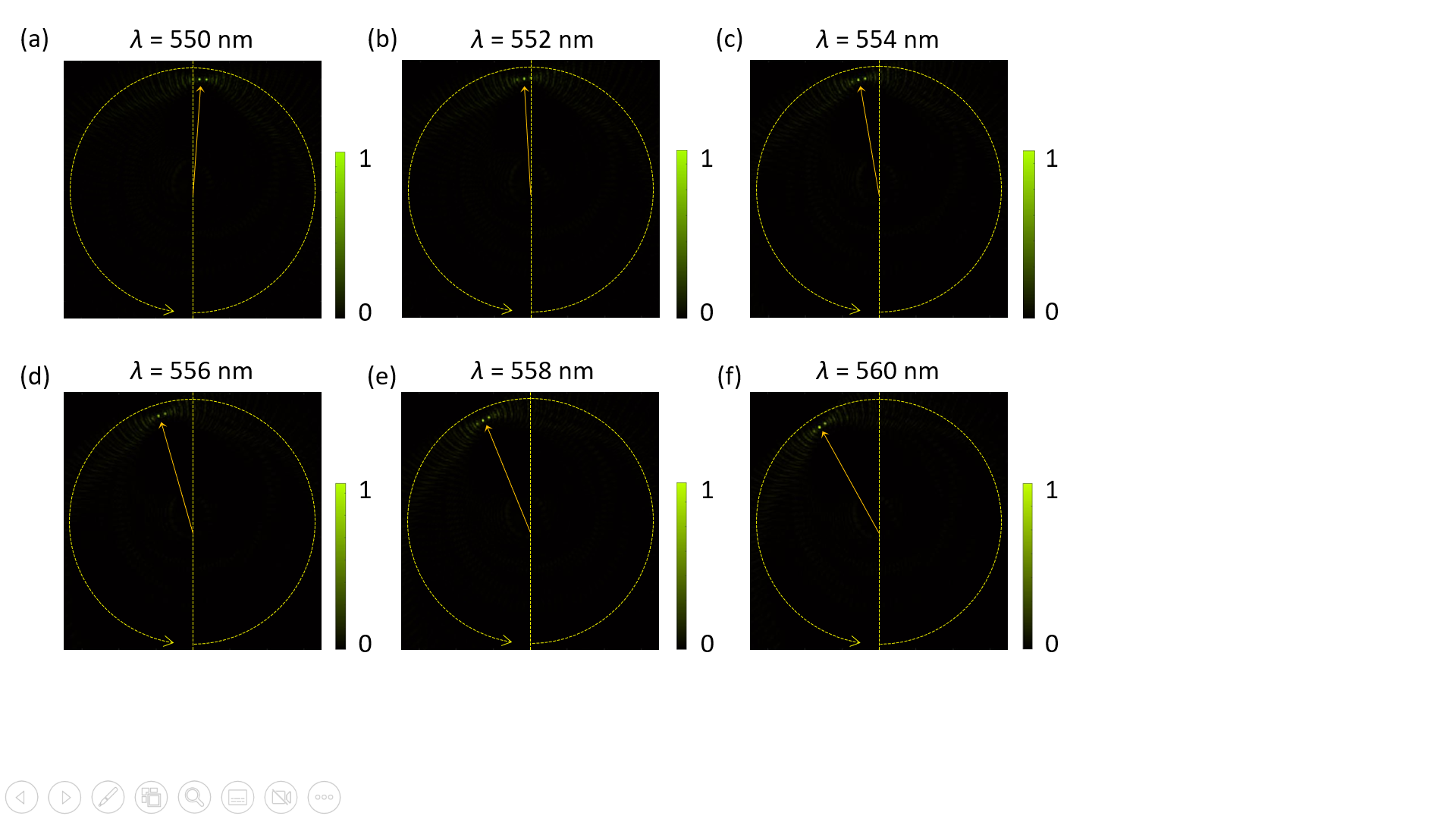


Fig. S15. Effect of small variation in the incident wavelength on the focal point.

**References**

1. Intaravanne, Y. *et al.* Color-selective three-dimensional polarization structures. *Light Sci Appl* **11**, 1–10 (2022).

2. Li, Y. *et al.* Longitudinally variable 3D optical polarization structures. *Sci Adv* **9**, eadj6675 (2023).

3. Arbabi, A., Horie, Y., Ball, A. J., Bagheri, M. & Faraon, A. Subwavelength-thick lenses with high numerical apertures and large efficiency based on high-contrast transmitarrays. *Nat Commun* **6**, 1–6 (2015).

4. Liang, H. *et al.* Ultrahigh Numerical Aperture Metalens at Visible Wavelengths. *Nano Lett* **18**, 4460–4466 (2018).

5. Zhuang, Z. P., Chen, R., Fan, Z. Bin, Pang, X. N. & Dong, J. W. High focusing efficiency in subdiffraction focusing metalens. *Nanophotonics* **8**, 1279–1289 (2019).

6. Wang, R. *et al.* Metalens for Generating a Customized Vectorial Focal Curve. *Nano Lett* **21**, 2081–2087 (2021).

7. Wang, R. *et al.* Compact multi-foci metalens spectrometer. *Light Sci Appl* **12**, 1–10 (2023).
